# Efficacy and safety of analgosedation with dexmedetomidine in critically ill mechanically ventilated children: a systematic review and meta-analysis of randomized controlled trials

**DOI:** 10.1007/s44253-025-00091-4

**Published:** 2025-09-24

**Authors:** David J. Zorko, Jennifer A. Klowak, Michael Vu, Yen-Mei Z. Mayer, Alexandra Pysklywec, Kimberley Lewis, Karen Choong

**Affiliations:** 1 Division of Pediatric Critical Care, Department of Pediatrics, McMaster University, 1280 Main St W, Room HSC-3E20, Hamilton, ON L8S 4K1 Canada; 2 Division of Critical Care, Department of Pediatrics, Children’s Hospital of Eastern Ontario, Ottawa, Canada; 3 Department of Pediatrics, Alberta Children’s Hospital, Calgary, Canada; 4 Michael G. DeGroote School of Medicine, McMaster University, Hamilton, Canada; 5 School of Medicine, Royal College of Surgeons, Dublin, Ireland; 6 Department of Public Health Sciences, Queen’s University, Kingston, Canada; 7 Department of Medicine, McMaster University, Hamilton, Canada; 8 Department of Health Research Methods, Evidence, and Impact, McMaster University, Hamilton, ON Canada

**Keywords:** Critical care, Pediatrics, Intensive care units, Dexmedetomidine, Sedation

## Abstract

**Objective:**

Dexmedetomidine is an increasingly popular analgosedative in critically ill children receiving invasive mechanical ventilation (IMV). We conducted a systematic review to evaluate the efficacy of dexmedetomidine compared to other analgosedatives in this population.

**Data sources:**

Seven electronic databases and trial registries to July 2024, without language restrictions.

**Study selection:**

Randomized controlled trials comparing dexmedetomidine to other analgosedatives in critically ill children receiving IMV.

**Data extraction and synthesis:**

Independently and in duplicate, we conducted data extraction, risk of bias assessment, and certainty assessment using Grading of Recommendations, Assessment, Development, and Evaluation. We conducted random-effects meta-analyses, calculating pooled risk ratios (RRs) and mean differences (MDs) with 95% confidence intervals.

**Results:**

We identified 12 trials (*n* = 592 patients). Pooled analyses demonstrated dexmedetomidine has little to no effect on IMV duration (MD -2.2 h [-3.3, -1.1]; moderate certainty), clinically important bradycardia (RR 1.42 [0.45, 4.49]; moderate certainty), or clinically important hypotension (RR 1.35 [0.48, 3.82]; moderate certainty). Dexmedetomidine may reduce delirium risk (RR 0.83 [0.64, 1.07]; low certainty), but impact on withdrawal is uncertain (RR 0.93 [0.55, 1.59]; very low certainty). A narrative synthesis was used to evaluate dexmedetomidine sedation efficacy, demonstrating very low certainty in attaining sedation target. One trial reported on long-term outcomes.

**Conclusions:**

Twelve trials evaluating dexmedetomidine have been conducted to date, with low or very low certainty for its impact upon delirium, withdrawal, and long-term outcomes. Future analgosedation trials require attention to intervention design, outcome selection and reporting to improve certainty in critical outcomes.

**Supplementary Information:**

The online version contains supplementary material available at 10.1007/s44253-025-00091-4.

## Background

Optimizing sedation in critically ill children receiving invasive mechanical ventilation (IMV) in the pediatric intensive care unit (PICU) is a challenging balance of avoiding adverse effects of both inadequate comfort and excessive sedation. Inadequately sedated patients may experience pain, anxiety, ventilator dyssynchrony, and are at risk of medical device dislodgement [1]. Excessive sedation may lead to delayed IMV liberation, withdrawal syndromes, delirium, potential hemodynamic instability and long-term neurocognitive or psychological sequelae [1–3]. Opioids and benzodiazepines are currently the most commonly used sedatives in the PICU [4–6] despite consistent evidence supporting their association with adverse sedation-related outcomes [7, 8]. Hence, there is great interest in alternative agents.

Dexmedetomidine is a highly selective α2-adrenergic agonist with sedative, anxiolytic, and mild analgesic properties, that is increasingly used in critically ill children [9–11]. Dexmedetomidine may enable reduction in opioid and benzodiazepine administration [11–13], thereby potentially mitigating delirium and withdrawal syndromes [11, 14]. By preserving respiratory drive, dexmedetomidine may facilitate quicker weaning from IMV [9, 15]. These proposed benefits have made dexmedetomidine an attractive analgosedative in the PICU. Current practice guidelines suggest the use of dexmedetomidine as the primary sedative agent in critically ill pediatric patients but acknowledge the low quality of evidence supporting this conditional recommendation [16, 17]. Dexmedetomidine’s efficacy for maintenance analgosedation compared to traditional agents remains unclear.

The aims of this systematic review were to evaluate efficacy and safety of intravenous (IV) dexmedetomidine in critically ill children receiving IMV compared to other analgosedatives. Our outcomes of interest were: (1) Adequacy of sedation; (2) Impact upon sedative burden; (3) PICU-related outcomes such as IMV duration and length of stay (LOS); (4) Sedation-related adverse events including withdrawal and delirium, and; (5) Long-term outcomes.

## Methods

We designed and registered this systematic review protocol a priori (PROSPERO CRD42021240041; March 31, 2021).

### Eligibility criteria

We included RCTs in children receiving IMV admitted to any type of PICU, that compared IV dexmedetomidine as a maintenance sedative to any other IV analgosedative(s) or placebo. Eligible studies had to report on at least one of the following outcomes of interest: (1) Adequacy of sedation, defined as the ability to achieve a *sedation target* as assessed by a sedation scale; (2) *Sedative burden*, defined as any measurement of total or cumulative weight-adjusted sedatives administered; (3) *Rate of delirium*, at any time measured by any delirium scoring tool; (4) *Rate of withdrawal*, at any time measured by any withdrawal scoring tool; (5) *Duration of IMV*; (6) *Time to extubation* after analgosedative cessation; (7) *Adverse events*, including unplanned extubation, hypotension, bradycardia, and clinically important hypotension and bradycardia (i.e. requiring sedation medication suspension, discontinuation or reduction, or additional interventions); (8) PICU or hospital *LOS*; and (9) *Long-term outcomes* (any) after PICU discharge.

We excluded studies that compared different doses of dexmedetomidine, or dexmedetomidine with another α2-agonist (e.g., clonidine). Studies evaluating dexmedetomidine for other indications (e.g., premedication, procedural sedation, arrhythmia management) were excluded, with no other restrictions on intervention or control sedative dose, duration, or use in combination with other sedatives.

### Database search

We searched the following databases and trial registries to January 25, 2021, without language restriction: Medline, Embase, CINAHL, Cochrane CENTRAL, Web of Science, ClinicalTrials.gov and PICUtrials.net. We updated the search on July 3, 2024. The search strategy was designed in consultation with a health research librarian, developed in Medline, and translated into the other databases (Online Resource 1). We reviewed reference lists of included trials and relevant literature reviews for records that potentially evaded the database search.

### Citation screening and data extraction

We exported records into Covidence software (Veritas Health Innovation, Melbourne, Australia). Screening was performed independently and in duplicate by two reviewers in two steps (title and abstract, then full text) against study selection criteria. After full text review, we confirmed eligibility of all retained citations. Two reviewers, independently and in duplicate, extracted data from included studies using piloted data extraction forms. Disagreements in screening or data extraction were resolved through discussion and consensus, or through arbitration by a third reviewer if necessary.

We contacted five corresponding authors for missing or ambiguous data required for our meta-analyses, of which two authors responded. We considered the potential impact of missing data on reported trial results in risk of bias (ROB) assessments.

### Risk of bias and certainty of evidence assessments

We assessed ROB independently and in duplicate using the Cochrane ROB2 tool [18]. The overall ROB for each trial was summarized ‘low’ if ROB was low in all domains, ‘some concerns’ if ROB was judged to raise some concerns in at least one domain and with no high-risk domains, or ‘high’ if ROB was high in at least one domain or ‘some concerns’ in multiple domains. We resolved disagreements by discussion and consensus.

We assessed the certainty of the aggregate body of evidence for each outcome using the Grading of Recommendations Assessment, Development and Evaluation (GRADE) approach [19], which classifies certainty as very low, low, moderate, or high based on evaluation of ROB, inconsistency, indirectness, imprecision, and publication bias. For outcomes with a narrative summary of the effect across trials, we used the GRADE approach published by Murad et al. to rate evidence certainty [20]. We reported results using GRADE narrative statements, using “likely/probably” to describe moderate certainty outcomes, “suggests/may” for low certainty, and “uncertain” for very low certainty [21].

### Data analysis

We planned to evaluate sedation target as the proportion of sedation scores within the sedation target defined in the study protocol. For purposes of comparison, we planned to report sedation burden across studies by converting to a consistent unit of analysis (i.e. mcg or mg) in cumulative weight-adjusted drug amounts.

We performed DerSimonian and Laird random-effects meta-analyses using RevMan 5.4 software (Cochrane Collaboration, Oxford, United Kingdom) to pool weighted effect estimates. We calculated pooled risk ratios (RRs) and risk differences (RD) for dichotomous outcomes, and mean differences (MDs) for continuous outcomes, with a corresponding 95% confidence intervals (CIs). We used the Mantel-Haenszel method to generate study weights for dichotomous outcomes due to small event counts [22], and the inverse variance method for continuous outcomes. We transformed nonparametric data to a mean and standard deviation assuming a normal distribution [23]. All outcomes had results from < 10 trials, precluding our planned publication bias assessment by funnel plot.

We assessed heterogeneity by visual inspection of forest plots, the Chi^2^ test (*p* < 0.10 representing significant heterogeneity), and the I^2^ statistic. We planned the following subgroup analyses: (1) Sedative comparator versus placebo comparator hypothesizing improved outcomes with dexmedetomidine when compared to placebo; (2) Dexmedetomidine used as a first-line analgosedative versus as an adjunct with other drug classes hypothesizing improved outcomes with dexmedetomidine as a first-line analgosedative; and (3) Cardiac surgical patients versus medical and non-cardiac surgical patients hypothesizing improved outcomes in cardiac surgical patients receiving dexmedetomidine. We planned a priori sensitivity analyses based on ROB hypothesizing improved outcomes with dexmedetomidine in some concerns/high ROB studies.

## Results

**Fig. 1:**
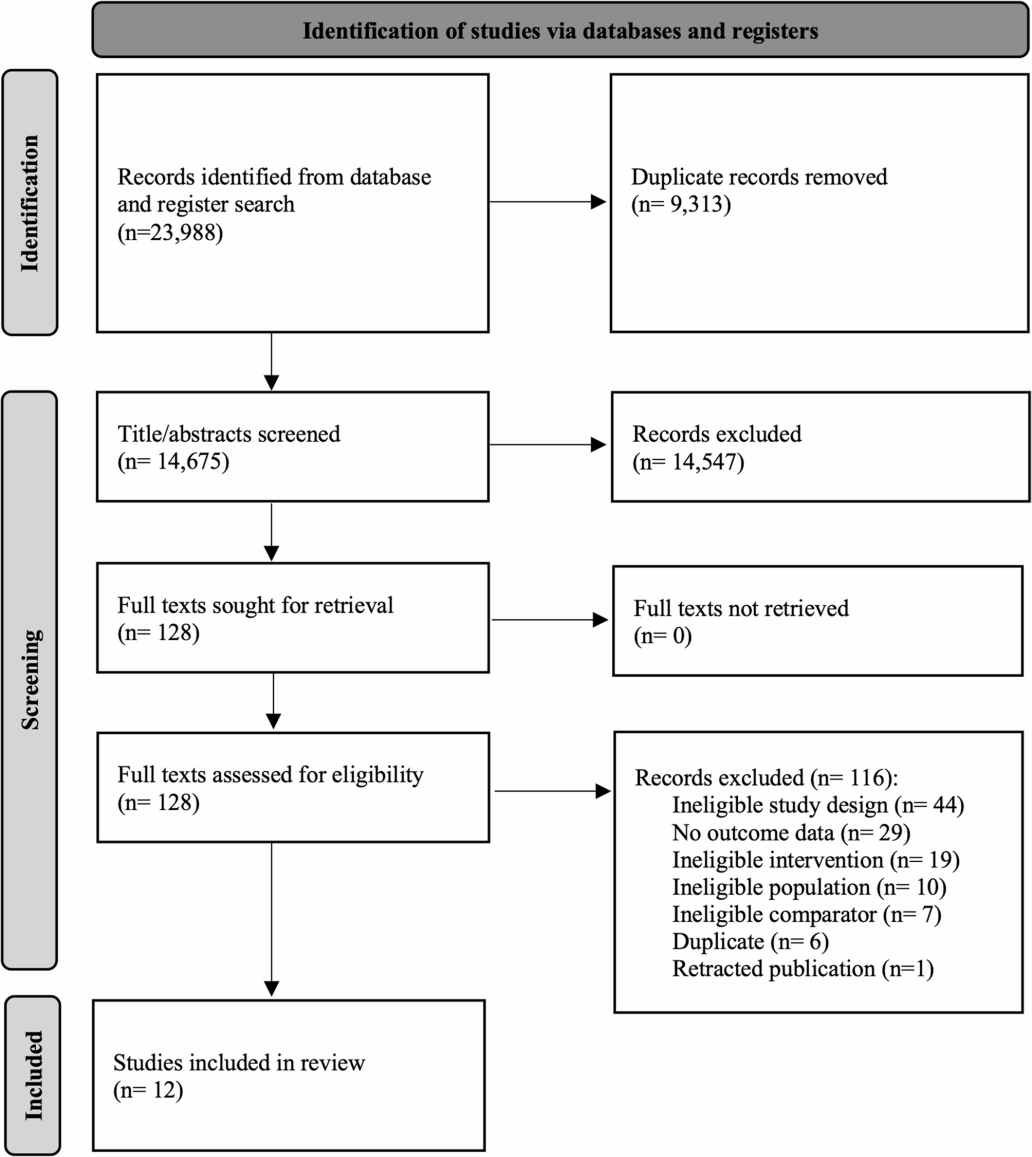
PRISMA flow diagram. Legend: Details of the citation search and screening process in this systematic review

Our search identified 14,675 unique citations of which 128 full text articles were assessed for eligibility and 12 unique RCTs were included in our analysis (Fig. 1). One additional RCT enrolling 200 children was identified in our original search, but subsequently excluded from our analyses due to retraction of publication [24].

### Characteristics of included trials

Characteristics of included trials are summarized in Table 1. All 12 studies were individually randomized, parallel group RCTs [12, 25–35], of which 10 were of a two-arm design. Two RCTs were of a three-arm design, comparing two different dexmedetomidine infusion doses to a single comparator arm [26, 35]. Three trials were multicentered, enrolling participants from the United States (*n* = 13 centres), Australia (*n* = 6 centres), or Italy (*n* = 3 centres). All trials had small sample sizes ranging from 30 to 66. Explicit funding statements were described in nine trials, and six reported a priori trial registration.

**Table 1:** Baseline characteristics of included studies

Trial characteristics	Participant characteristics^a^	Intervention group(s)	Control group(s)	Trial sedation target	Initiation of DXM infusion	Duration of DXM infusion
Becker et al. (2023) United States Multi-centered *n* = 30, blinded	Mean age: 1.5 years % Female: 40% Admission diagnosis: 100% Medical-surgical	Group 1: DXM 0.5 mcg/kg bolus, then 0.2 mcg/kg/hr + fentanyl usual care dosing	Placebo + fentanyl usual care dosing	SBS or RASS − 1 or lower	Within 2 h of randomization after > 24 h of IMV	Until discontinued or at extubation, up to 7 days
		Group 2: DXM 0.5 mcg/kg bolus, then 0.5 mcg/kg/hr + fentanyl usual care dosing				
Long et al. (2023) Australia Single-centered *n* = 66, blinded	Mean age: 0.3 years % Female: 43.9% Admission diagnosis: 100% CV surgical	DXM 0.25–1.25 mcg/kg/hr	Midazolam 0.02–0.08 mg/kg/hr	SBS − 1 to + 1	Cardiopulmonary bypass start	Until discontinued or up to 14 days
Attia et al. (2022) Egypt Single-centered *n* = 60, blinded	Mean age: 2.2 years % Female: 61.7% Admission diagnosis: 100% CV surgical	DXM 0.5 mcg/kg bolus, then 0.2–1.5 mcg/kg/hr	Fentanyl 0.5 mcg/kg bolus, then 1–3 mcg/kg/hr	COMFORT-B, target NR	NR	NR
Gulla et al. (2021) India Single-centered *n* = 49, open label	Mean age: 0.9 years % Female: 51% Admission diagnosis: 100% Medical-surgical	DXM 0.25 mcg/kg/hr + fentanyl 1 mcg/kg bolus and midazolam 0.1 mg/kg bolus	Midazolam 0.1 mg/kg bolus then 1 mcg/kg/min + fentanyl 1 mcg/kg bolus	PSCH sedation scale level 4 to 5	At randomization	At extubation or up to 7 days
Mondardini et al. (2021) Italy Multi-centered *n* = 45, blinded	Mean age: 1.2 years % Female: 35.6% Admission diagnosis: 100% Medical-surgical	DXM 0.2–0.4 mcg/kg/hr (age-dependant)	Placebo	NR	Within 24 h of weaning opioids and/or benzodiazepines of ≥ 5 days duration	Weaned to off after opioids and benzodiazepines discontinued
Erickson et al. (2020) Australia Multi-centered *n* = 60, open label	Mean age: 0.9 years % Female: 43.8% Admission diagnosis: 80% Medical-surgical, 20% CV surgical	DXM 1.0 mcg/kg/hr	Usual care	SBS − 1 to + 1	Within 12 h of IMV	Until discontinued or up to 14 days
Garisto et al. (2018) Italy Single-centered *n* = 60, open label	Mean age: 0.5 years % Female: 38.3% Admission diagnosis: 100% CV surgical	DXM 0.5 mcg/kg/hr + midazolam 0.05 mg/kg/hr + morphine 10 mcg/kg/hr	Midazolam 0.1 mg/kg/hr + morphine 20 mcg/kg/hr	COMFORT scale 17 to 26	PICU arrival	Until surgical drains removed then weaned off over 8 h
Saleh et al. (2015) Egypt Single-centered *n* = 50, blinded	Mean age: 5.9 years % Female: 54% Admission diagnosis: 100% Non-CV surgical	DXM 0.3 mcg/kg/hr	Fentanyl 1.0 mcg/kg/hr	RSS 4 to 5	PICU arrival	18 h
Aydogan et al. (2013) Turkey Single-centered *n* = 32, blinded	Mean age: 14.2 years % Female: 46.9% Admission diagnosis: 100% Non-CV surgical	DXM 0.25 mcg/kg bolus (optional), then 0.4 mcg/kg/hr	Midazolam 0.1 mg/kg bolus, then 0.1 mg/kg/hr	RASS − 2 to + 1	Immediately post-op	Up to 24 h or until extubation
Hussein et al. (2013) Egypt Single-centered *n* = 50, blinded	Mean age: 6 years % Female: 48% Admission diagnosis: 100% Non-CV surgical	DXM 0.4 mcg/kg/hr	Fentanyl 1 mcg/kg/hr	NR	PICU arrival	Until extubation
Prasad et al. (2012) India Single-centered *n* = 60, blinded	Mean age: 5.9 years % Female: 46.7% Admission diagnosis: 100% CV surgical	DXM 0.5 mcg/kg/hr	Fentanyl 1.0 mcg/kg/hr	NR	PICU arrival	Until extubation
Tobias et al. (2004) United States Single-centered *n* = 30, open label	Mean age: 3.3 years % Female: 33.3% Admission diagnosis: NR	*Group 1*: DXM 0.25 mcg/kg bolus (optional), then 0.25 mcg/kg/hr	Midazolam 0.1 mg/kg bolus (optional), then 0.1 mg/kg/hr	NR	At intubation	Up to 24 h
		*Group 2*: DXM 0.5 mcg/kg bolus (optional), then 0.5 mcg/kg/hr				

^a^Medical-surgical includes non-CV surgical patients. CV surgical patients are specifically described, where applicable

*CV* indicates cardiovascular, *DXM* dexmedetomidine, *IMV* invasive mechanical ventilation, *NR* not reported, *PSCH* Penn State Children’s Hospital, *RASS* Richmond Agitation-Sedation Scale*RSS* Ramsay sedation score*, SBS *State behavioural scale

#### Participants

A total of 592 participants were enrolled across trials with a mean (SD) age of 3.6 (4.0) years, of which 51.7% were male. Six trials reported on severity of illness measurements, using different scoring tools. Overall, 304 (51.4%) participants had medical and non-cardiac surgical diagnoses, 258 (43.6%) had cardiac surgical diagnoses, and 30 (5.1%) were unspecified. Seven trials (58.3%) enrolled exclusively post-operative patients [12, 25, 28, 30, 31, 33, 34], of which four enrolled exclusively post-cardiac surgery patients [25, 28, 31, 33].

#### Interventions

The intervention protocols of the included trials were heterogeneous.Nine trials (75.0%) used dexmedetomidine as the first-line agent, allowing use of additional analgosedatives if dexmedetomidine alone did not achieve the targeted sedation goal either in the form of infusions (n=2) [27, 31] or as rescue boluses only (n=7) [12, 25, 29, 30, 33–35]. In the remaining three RCTs, dexmedetomidine was not the first-line analgosedative agent. Two trials administered a dexmedetomidine infusion in combination with other analgosedative infusions [26, 28], and one trial added dexmedetomidine as an adjunct to other analgosedative infusions after ≥5 days of use to facilitate analgosedative weaning [32].

Dexmedetomidine dosing and titration was very heterogenous across the included trials. In eight trials (66.7%) dexmedetomidine infusion was initiated without an initial bolus, while two trials mandated a 0.5 mcg/kg bolus at infusion initiation [25, 26]. Two trials allowed for dexmedetomidine boluses as needed (0.25 or 0.5 mcg/kg) [12, 35]. Most trials (*n* = 11, 91.7%) did not report a mean or median administered dexmedetomidine infusion dose. Starting dexmedetomidine infusion dose was most commonly less than 0.5 mcg/kg/hr (*n* = 9 trials, 75.0%), with a range of 0.2–1 mcg/kg/hr. The maximum dexmedetomidine infusion dose was variable and ranged from 0.2 to 1.6 mcg/kg/hr across all trials. Five trials (41.7%) used a maximum dose ≤ 0.5 mcg/kg/hr [26, 28, 30, 33, 34], three trials (25.0%) used maximum doses ≥ 1 mcg/kg/hr [25, 27, 31], and one trial (8.3%) used a maximum dose of 0.75 mcg/kg/min [29]. One additional trial reported using different maximum doses for neonates (0.8 mcg/kg/min) and children (1.6 mcg/kg/hr) [32]. Two trials (16.7%) did not report the maximum infusion dose used [12, 35].

Seven trials (58.3%) permitted the dexmedetomidine infusion to be titrated during the study [12, 25, 27, 29, 31, 32, 35], and the remaining five trials used a fixed dexmedetomidine infusion dose ranging from 0.2 to 0.5 mcg/kg/hr. In five trials (41.7%) dexmedetomidine was infused for ≤ 24 h and > 24 h in another five trials. Two trials (16.7%) did not report actual infusion duration.

#### Comparators

Dexmedetomidine was compared to midazolam in four trials (33.3%) [12, 29, 31, 35], opioids in four (33.3%) [25, 30, 33, 34], placebo (with or without other permissible analgosedatives) in two (16.7%) [26, 32], a combination of midazolam and morphine in one (8.3%) [28], and “usual care” (described as any sedatives, excluding α2-agonists, at the discretion of the treating clinician) in one trial (8.3%) [27].

### Risk of bias (ROB)

Overall ROB of each study with the corresponding outcome of interest are summarized in Online Resource 2. Across all trials, most outcomes were assessed as having some concerns or high ROB (60.0%; *n* = 27/45 total outcomes). This was commonly due to inadequate randomization processes, lack of blinding, inadequate cointervention reporting, and/or reporting of trial results. As only one trial reported on any long-term outcome after PICU discharge, we did not assess ROB or certainty of evidence for this outcome.

### Effects of interventions

A summary of findings for key outcomes is detailed in Table 2. A full evidence profile is provided in Online Resource 3.

**Table 2:** GRADE summary of findings

Outcomes	No. of Patients		Effect		Certainty	Narrative Summary
No. of Trials	DXM	Control	Relative (95% CI)	Absolute (95% CI)		
Sedation target						
3	Three trials (*n* = 170 participants) assessed sedation efficacy. Two trials found that DXM, compared to other sedatives, increased the portion of sedation assessments within sedation target. However, CIs in one trial included both substantially higher, and a possibility of less, assessments within sedation target. A third trial found that DXM, compared to midazolam, decreased time and proportion of assessments within sedation target.				⨁◯◯◯ Very low	DXM has an uncertain effect on sedation assessments within target in critically ill children receiving IMV when compared to other analgosedatives.
Duration of mechanical ventilation						
6	148	132	–	MD 2.18 h lower (3.28 lower to 1.09 lower)	⨁⨁⨁◯ Moderate	DXM probably results in little to no difference in IMV duration in critically ill children when compared to other analgosedatives.
Delirium						
3	29/68 (42.6%)	33/58 (56.9%)	RR 0.83 (0.64–1.07)	97 fewer per 1000 (205 fewer to 40 more)	⨁⨁◯◯ Low	DXM may reduce incidence of delirium in critically ill children receiving IMV when compared to other analgosedatives.
Withdrawal						
3	19/81 (23.5%)	23/82 (28.0%)	RR 0.93 (0.55–1.59)	20 fewer per 1000 (126 fewer to 165 more)	⨁◯◯◯ Very low	DXM has an uncertain effect on incidence of withdrawal in critically ill children receiving IMV when compared to other analgosedatives.
Clinically important bradycardia						
10	13/255 (5.1%)	3/228 (1.3%)	RR 1.42 (0.45–4.49)	6 more per 1000 (7 fewer to 46 more)	⨁⨁⨁◯ Moderate	DXM probably results in little to no difference in risk of bradycardia requiring intervention in critically ill children receiving IMV when compared to other analgosedatives.
Clinically important hypotension						
8	13/206 (6.3%)	3/179 (1.7%)	RR 1.35 (0.48–3.82)	6 more per 1000 (9 fewer to 47 more)	⨁⨁⨁◯ Moderate	DXM probably results in little to no difference in risk of hypotension requiring intervention in critically ill children receiving IMV when compared to other analgosedatives.
PICU length of stay						
7	166	154	–	MD 0.2 days fewer (0.35 fewer to 0.05 fewer)	⨁⨁⨁◯ Moderate	DXM probably results in little to no difference in PICU length of stay in critically ill children receiving IMV when compared to other analgosedatives.

*DXM* indicates dexmedetomidine,* IMV* invasive mechanical ventilation, *MD* mean difference, *RR* risk ratio

#### Sedation efficacy

Sedation target was heterogeneously reported in three trials (*n* = 170 participants; Online Resource 4) [27, 29, 31]. Our pre-specified meta-analysis could not be performed and we conducted a narrative synthesis. The reported sedation efficacy of dexmedetomidine was variable in the three trials, compared to other sedatives. Two trials found that dexmedetomidine increased the portion of sedation assessments within sedation target. However, CIs of the effect estimate in one trial included both substantially higher, and a possibility of less assessments within sedation target. The remaining trial found that dexmedetomidine decreased time and proportion of assessments within sedation target, compared to midazolam. Overall, the evidence is very uncertain about the effect of dexmedetomidine on time within sedation target because of ROB, imprecision, and inconsistency in trial results (Table 2).

Five trials (*n* = 231 participants) reported on sedation burden with weight-adjusted analgosedative doses [27, 28, 31, 32, 35]. We could not perform meta-analysis or examine certainty in evidence due to important heterogeneity and inability to reconcile a measurement of overall sedative burden across multiple agents. No trials administered the same cointervention drug classes. We therefore summarize the results of these trials descriptively in Online Resource 5.

#### IMV duration

**Fig. 2:**
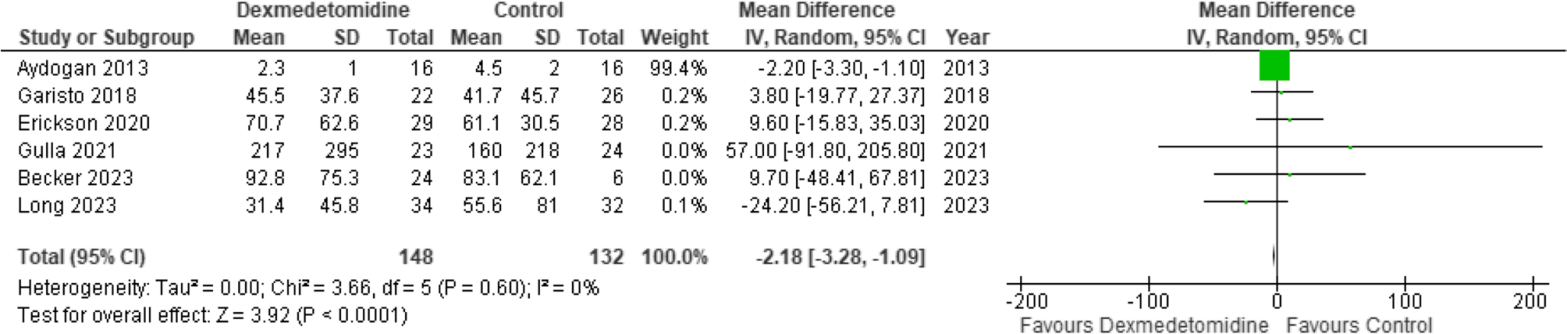
Forest plot for duration of mechanical ventilation (hours).Legend: df, indicates degress of freedom; IV, inverse variance (random effects model)

The pooled estimate across six RCTs (*n* = 280 participants) [12, 26–29, 31] showed that dexmedetomidine likely results in little to no difference in IMV duration compared to other sedation strategies (MD −2.2 h, 95% CI −3.3 to −1.1; moderate certainty; Fig. 2; Table 2).

#### Time to extubation

Based on four trials comparing dexmedetomidine to fentanyl for post-surgical analgosedation (*n* = 220 participants) [25, 30, 33, 34], dexmedetomidine reduced the time to extubation but the evidence is very uncertain due to ROB, inconsistency, and imprecision (MD −3.0 h, 95% CI −4.9 to −1.1; very low certainty; Online Resource 6; Online Resource 3).

#### Delirium

**Fig. 3:**
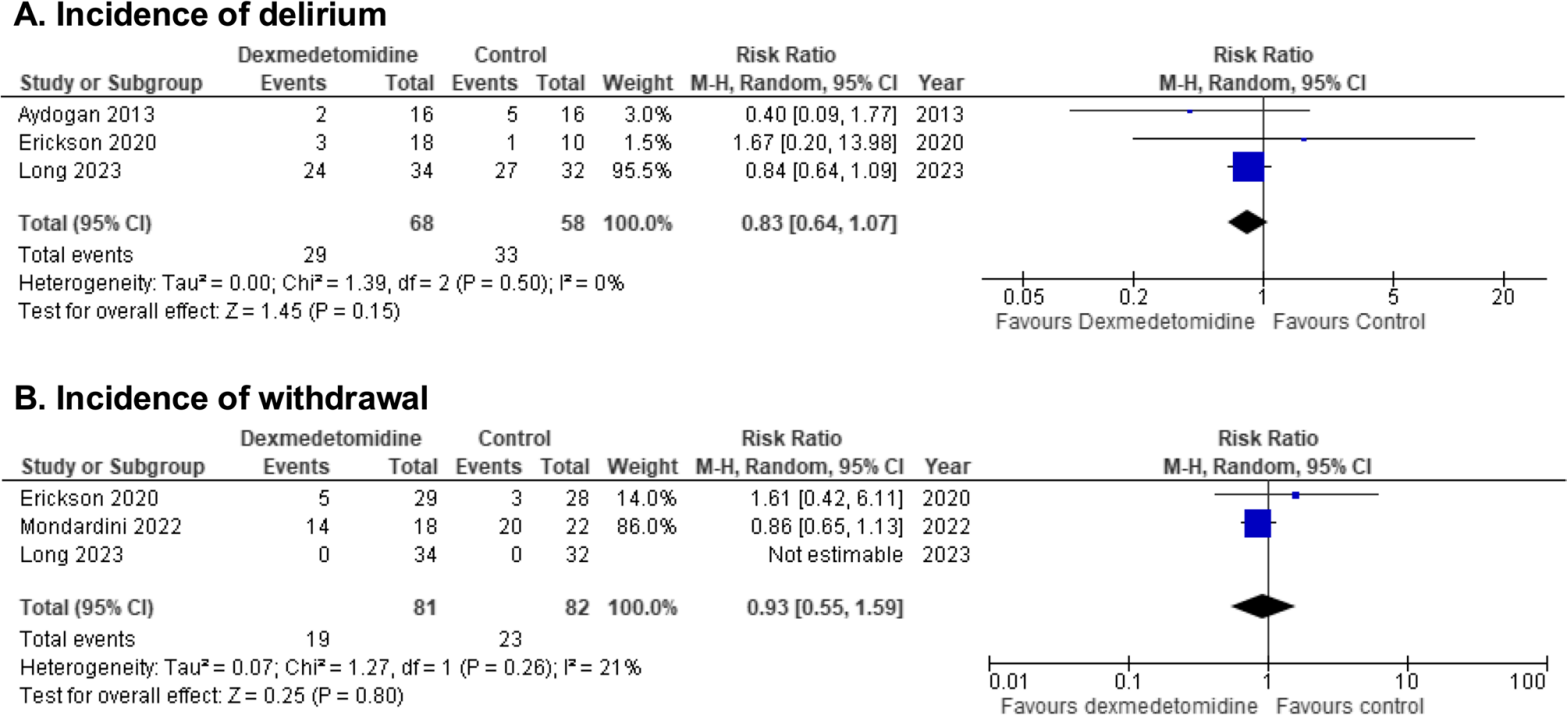
Forest plots for A. Incidence of delirium, and B. Incidence of withdrawal. Legend: df, indicates degress of freedom; M-H, Mantel-Haenszel (random effects model)

The pooled estimate from three RCTs (*n* = 126 participants) that compared dexmedetomidine to midazolam (*n* = 2 trials) [12, 31] or usual care (*n* = 1 trial) [27] showed that dexmedetomidine may reduce the risk of delirium (RR 0.83, 95% CI 0.64 to 1.07; RD 97 fewer per 1000, 95% CI 205 fewer to 40 more; low certainty; Fig. 3; Table 2).

#### Withdrawal

Three trials (*n* = 163 participants) examined incidence of withdrawal using the Withdrawal Assessment Tool-1 [27, 31, 32]. Dexmedetomidine, when compared to other analgosedatives, had an uncertain impact upon withdrawal (RR 0.93, 95% CI 0.55 to 1.59; RD 20 fewer per 1000, 95% CI 126 fewer 165 more; very low certainty; Fig. 3; Table 2).

#### Adverse events

**Fig. 4:**
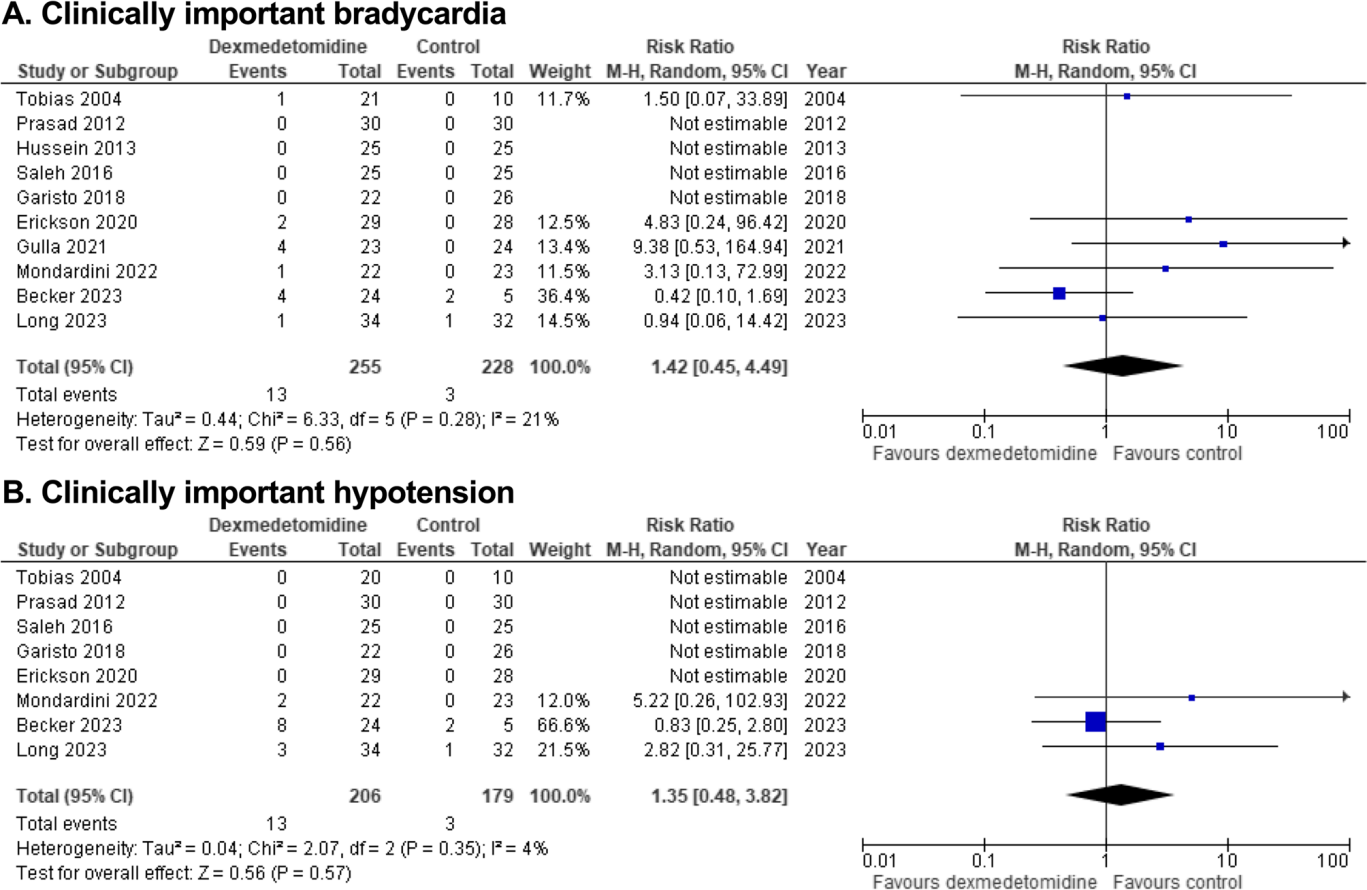
Forest plots for A. Clinically important bradycardia, and B. Clinically important hypotension. Legend: df, indicates degress of freedom; M-H, Mantel-Haenszel (random effects model)

Study author definitions of adverse events are summarized in Online Resource 7. Eight trials (*n* = 345 participants) [12, 26–29, 31, 32, 35] reported that dexmedetomidine may increase bradycardia compared to other analgosedatives (RR 2.45, 95% CI 0.90 to 6.65; RD 62 more per 1000, 95% CI 4 fewer to 241 more; low certainty; Online Resource 6; Online Resource 3). The overall rate of clinically important bradycardia in participants receiving dexmedetomidine was low (12/255; 5.1%). Pooled analysis from 10 trials (*n* = 483 participants) [26–35] suggests that dexmedetomidine probably results in little to no difference in bradycardia requiring intervention (RR 1.42, 95% CI 0.45 to 4.49; RD 6 more per 1000, 95% CI 7 fewer to 46 more; moderate certainty; Fig. 4; Table 2).

Nine trials (*n* = 445 participants) [25–28, 31–35] examined incidence of hypotension. Dexmedetomidine resulted in little to no effect on hypotension compared to other analgosedatives (RR 1.99, 95% CI 0.80 to 4.96; RD 19 more per 1000, 95% CI 4 fewer to 7.6 more; low certainty; Online Resource 6; Online Resource 3). The overall rate of clinically important hypotension in participants receiving dexmedetomidine was low (13/206; 6.3%). Pooled analysis from eight trials (*n* = 385 participants) [26–28, 31–35] demonstrated that dexmedetomidine probably results in little to no difference in hypotension requiring intervention (RR 1.35, 95% CI 0.48 to 3.82; RD 6 more per 1000, 95% CI 9 fewer to 47 more; moderate certainty; Fig. 4; Table 2).

Unplanned extubation was reported in five RCTs (*n* = 269 participants), of which four reported no overall events [27, 30, 33, 34] and one reported data as part of a composite outcome [29].

#### Length of stay

Seven trials reported on PICU LOS (*n* = 320 participants) [12, 26–29, 31, 32]. Sedation with dexmedetomidine resulted in little to no difference in PICU LOS compared with other sedation strategies (MD −0.20 days, 95% CI −0.35 to −0.05 days; moderate certainty; Online Resource 6; Table 2). The pooled estimate from three trials (*n* = 170 participants) [27, 29, 31] showed that dexmedetomidine resulted in little to no difference in hospital LOS (MD 0.53 days, 95% CI −2.97 to 4.03 days; low certainty; Online Resource 6; Online Resource 3).

#### Long-term outcomes

One trial (*n* = 47 participants) [31] comparing dexmedetomidine to midazolam reported on outcomes at 12-months post-PICU discharge and found no impact on overall quality-of-life (using the Pediatric Quality of Life Inventory), but a higher odds of scoring ≥ 2 SD below the mean in fine motor skills (OR 10.3; 95% CI 1.1, 93.2) as measured by the Ages and Stages Questionnaire in children who received dexmedetomidine.

### Subgroup and sensitivity analyses

Due to insufficient data, we were unable to conduct subgroup and sensitivity analyses across all outcomes of interest. Of subgroup and sensitivity analyses able to be performed, we did not find any subgroup effects (Online Resource 8). We did not find any differences in IMV duration, time to extubation, PICU LOS, hypotension or bradycardia in studies evaluating cardiac surgery patients compared to general medical-surgical patients.

## Discussion

This systematic review and meta-analysis of RCTs examining dexmedetomidine analgosedation in critically ill children receiving IMV reveals several key findings. First, only 12 RCTs, enrolling a total of 592 participants, have been conducted since 2004. All trials had small sample sizes, heterogeneous dexmedetomidine dosing protocols, and demonstrated limitations in reporting of intervention fidelity and patient-important outcomes. Second, this evidence suggests that while dexmedetomidine has an uncertain effect on achieving a sedation target and reducing rate of withdrawal compared to other analgosedatives, it may reduce the rate of delirium and probably results in little to no difference in IMV duration or incidence of clinically important hypotension and bradycardia. Third, there is a paucity of evidence concerning the impact of dexmedetomidine on long-term outcomes and cardiac surgical populations receiving IMV. ROB and imprecision reduced certainty of evidence in several outcomes.

Current guidelines suggest α2-adrenergic agonists as the primary sedative option in critically ill children [16, 17], and as a result, the use of dexmedetomidine is increasing [4, 9, 36]. As these recommendations were based on low quality evidence and the use of dexmedetomidine may be prohibited by cost or adverse effect concerns, it prompts close examination of more current evidence informing our analgosedation practices. While there are limited studies evaluating the efficacy of individual analgosedatives in critically ill children [16, 17, 37], their potential downstream neurotoxic impact is now well recognized including their association with post-PICU post-traumatic stress disorder, impairments in cognitive function, and reduced overall functioning [38–41]. Resultantly, analgosedation stewardship has become a central component of PICU liberation bundles, which aim to provide targeted analgosedation while minimizing iatrogenic harm and ultimately improve short and long-term patient outcomes [42, 43]. To this end, we found low certainty evidence suggesting that dexmedetomidine may reduce delirium; however, the 95% CI of the effect estimate includes the possibility of a trivial increase in delirium rates. When examining RDs in the face of low event rates, we also found moderate certainty evidence that dexmedetomidine likely does not increase clinically important bradycardia or hypotension (i.e. less than one more per 100 receiving dexmedetomidine).

The results of this systematic review should be interpreted with caution. We evaluated outcomes that we considered clinically important (e.g. attainment of sedation target, IMV duration) or patient important (e.g. delirium, long-term outcomes) and the collective evidence across trials demonstrated significant variability in the inclusion and reporting of these outcomes. Judgements of efficacy, or lack thereof, and some adverse events are based on low or very low certainty evidence. This systematic review reveals important ROB in trials due to issues with allocation concealment, blinding, and/or outcome assessment, which may have contributed to unbalanced administration of analgosedative co-interventions. The small number of trials with small sample sizes lead to imprecision in effect estimates, with CIs encompassing important benefits and harms. The small number of trials precluded performance of many pre-planned subgroup analyses, and thus we were not able to conclusively evaluate the efficacy of dexmedetomidine in pediatric cardiac surgical patients. Finally, important population characteristics were inconsistently reported across trials, specifically severity of illness and IMV duration.

Another key contributing factor to the lack of demonstrable efficacy for dexmedetomidine may be due to the considerable variability in dexmedetomidine dosing and titration across trials. Most trials used dosing regimens lower than reported in current practice [4, 36], large pediatric observational studies [9, 11, 44], and large adult RCT data [45]; these all report maximum dosing between 1 and 1.5 mcg/kg/hr. This may reflect growing experience with dexmedetomidine in the PICU over time and the influence of cost limitations [6] or regulatory bodies (e.g. US Food and Drug Administration [FDA]) on regional practices. Dexmedetomidine infusion is only FDA approved for adult ICU sedation (up to 0.7 mcg/kg/hr), sedation in non-invasive procedures for children aged > 1 month (up to 1.5 mcg/kg/hr), and currently not approved for PICU sedation [46]. Further, several trials enrolled exclusively post-operative patients whose typically shorter IMV duration has likely implications on analgosedative dosing, cumulative analgosedative burden, drug tolerance and its sequelae (e.g. withdrawal). The heterogeneous dexmedetomidine administration across trials may also explain why there was not more convincing evidence of clinically important hypotension and bradycardia. Analgosedation is a complex intervention that is titrated to variable endpoints by physicians, nurses, and pharmacists across a diverse population of critically ill medical and surgical patients. Intervention fidelity assessment is important for tailored and titrated interventions [47], but uncommonly reported in PICU analgosedation research [48]. In our systematic review, intervention fidelity was reported in only one trial. The reporting of complex analgosedative interventions and analgosedative burden is a priority area of improvement for future trials.

The findings of this systematic review are consistent with the prior PICU sedation evidence [48] demonstrating that trials seldom measure patient-important short- or long-term outcomes like delirium, withdrawal, function or cognition, resulting in lower certainty evidence. A recent survey of pediatric intensivists identified delirium and withdrawal as the highest priority outcomes for analgosedation trials, after duration of IMV [6]. Only one trial in our review reported on a long-term outcome after PICU discharge, yet these are critically important to clinicians and families [49]. PICU providers routinely encounter children with CNS dysmaturity, neurologic critical illness, and prescribe diverse analgosedatives for prolonged durations that expose patients to neurological stressors like delirium, withdrawal syndromes, and disrupted sleep architecture. Furthermore, the diverse population of critically ill children necessarily treated with analgosedatives for prolonged periods raises age-dependent issues of drug tolerance, dependency, delirium and drug withdrawal syndrome, underscoring the importance of measuring these sedation-related adverse effects [50, 51]. Methodologically rigorous RCTs are needed to evaluate analgosedative strategies and approaches that focus on not just clinically important efficacy outcomes, but on the harmful effects of sedatives (i.e. withdrawal, delirium, IMV duration, LOS) as well longer-term neurotoxic effects (i.e. neurocognitive and psychological outcomes) that are now recommended by core outcome sets [52].

Prior systematic reviews on this topic have important limitations. They have examined heterogeneous patient populations and applications of dexmedetomidine [53–56], have generated effect estimates pooling RCT and non-randomized study data [53–57], and have not consistently considered ROB or certainty of evidence in included studies [54–57]. This systematic review improves upon others in its comprehensive search strategy, prospectively registered protocol, adherence to methodological standards, and application of GRADE to evaluate certainty of evidence and contextualize results. Our review is the first to summarize sedation efficacy and patient-important outcomes (e.g., delirium, withdrawal, long-term outcomes), and reveals important methodological considerations related to patient population and intervention delivery that can improve future analgosedation trial design. We highlight reporting areas in need of attention, including lack of intervention fidelity, heterogeneous reporting of sedation efficacy, and lack of data on patient-important outcomes. These are also highlighted in recent consensus statements on pediatric analgosedation trials [58], signaling that the field requires a core outcome set and standards for intervention reporting. We included recent trials without restrictions on duration of IMV. In this way, our review provides a holistic evaluation of the many trials conducted and identifies their key challenges and limitations.

This review also has limitations. Our a priori protocol focused only on children receiving IMV, as we felt dexmedetomidine analgosedation in non-invasive ventilation was a unique population of its own specific merit. We were unable to perform pre-specified meta-analysis for sedation efficacy outcomes as significant heterogeneity in reported results precluded pooling data. These outcomes were therefore analyzed in a narrative manner. Many subgroup analyses could not be completed, and it is possible that specific subgroups were underpowered due to small number of contributing trails and participants. Subgroup results (e.g. cardiac surgery patients) should therefore be interpreted with caution. We were unable to examine funnel plots to detect small study effects, mitigating this by searching clinical trial registries and databases without language restrictions.

## Conclusion

Dexmedetomidine likely results in little to no difference in duration of IMV, PICU LOS, or clinically significant hypotension and bradycardia. There is a paucity of evidence evaluating dexmedetomidine’s impact upon delirium, withdrawal, and long-term outcomes. As the use of dexmedetomidine in critically ill children is expanding, future research is needed that reflects dosing currently used in clinical practice and includes core clinical and patient-important long-term outcomes. A specific reporting checklist (or adaptation of existing intervention checklists) and core outcome set for future analgosedation trial design would be beneficial.

## Supplementary Information

Supplementary material 1. Online Resource 1. Search strategies Online Resource 2. Risk of bias summary Online Resource 3. Evidence profile Online Resource 4. Sedation target outcome narrative summary Online Resource 5. Sedation burden outcome narrative summary Online Resource 6. Forest plots Online Resource 7. Summarized author definitions of adverse events Online Resource 8. Subgroup and sensitivity analyses

## Data Availability

All data supporting the findings of this study are available within the paper and its Online Resources.
