# Supplementary material for: Efficacy and safety of analgosedation with dexmedetomidine in critically ill mechanically ventilated children: a systematic review and meta-analysis of randomized controlled trials
Source: Intensive Care Med Paediatr Neonatal. 2025 Sep 24;3(1):30. doi: 10.1007/s44253-025-00091-4 (PMC12460513; doi:10.1007/s44253-025-00091-4)

**SUPPLEMENTARY INFORMATION**

**Efficacy and safety of sedation with dexmedetomidine in critically ill mechanically ventilated children: a systematic review and meta-analysis of randomized controlled trials**

David J. Zorko, MD MSc*^1^; Jennifer A. Klowak, MD MSc*^2,3^; Michael Vu, MD^4^; Yen-Mei Z. Mayer, MB BCh candidate^5^; Alexandra Pysklywec, BASc^6^; Kimberley Lewis, MD MSc^7,8^; and Karen Choong, BCh MB MSc^1,8^

*These authors contributed equally as first authors.

^1^ Division of Pediatric Critical Care, Department of Pediatrics, McMaster University, Hamilton, Canada

^2^ Division of Critical Care, Department of Pediatrics, Children’s Hospital of Eastern Ontario, Ottawa, Canada

^3^ Department of Pediatrics, Alberta Children’s Hospital, Calgary, Canada

^4^ Michael G. DeGroote School of Medicine, McMaster University, Hamilton, Canada

^5^ School of Medicine, Royal College of Surgeons, Dublin, Ireland

^6^ Department of Public Health Sciences, Queen's University, Kingston, Canada

^7^ Department of Medicine, McMaster University, Hamilton, Canada

^8^ Department of Health Research Methods, Evidence, and Impact, McMaster University, Hamilton, Ontario, Canada

**TABLE OF CONTENTS**

[Online Resource 1. Search strategies 2](#_Toc194659351)

[Online Resource 2. Risk of bias summary 6](#_Toc194659352)

[Online Resource 3. Evidence profile 8](#_Toc194659353)

[Online Resource 4. Sedation target outcome analysis^a^ 11](#_Toc194659354)

[Online Resource 5. Sedation burden outcome analysis^a^ 12](#_Toc194659355)

[Online Resource 6. Forest plots 13](#_Toc194659356)

[Online Resource 7. Summarized author definitions of adverse events 16](#_Toc194659357)

[Online Resource 8. Subgroup analyses 17](#_Toc194659358)

# Online Resource 1. Search strategies

**A. Medline (January 25, 2021)**

| **Line** | **Search** | **Result** |
| --- | --- | --- |
| 1 | exp Critical Care/ | 59428 |
| 2 | exp Critical Illness/ | 30683 |
| 3 | exp Intensive Care Units/ | 87957 |
| 4 | exp Respiration, Artificial/ | 79160 |
| 5 | acute care.mp. | 23298 |
| 6 | Critical care.mp. | 75294 |
| 7 | Critical* ill*.mp. | 63868 |
| 8 | ICU.mp. | 61662 |
| 9 | PICU.mp. | 4984 |
| 10 | Intensive care.mp. | 185230 |
| 11 | Intubat*.mp. | 87506 |
| 12 | Ventilat*.mp. | 194962 |
| 13 | Respiratory care unit*/mp. | 710 |
| 14 | 1 or 2 or 3 or 4 or 5 or 6 or 7 or 8 or 9 or 10 or 11 or 12 or 13 | 524626 |
| 15 | exp Pediatrics/ | 59282 |
| 16 | exp Child/ | 1940693 |
| 17 | exp Adolescent/ | 2063074 |
| 18 | exp Infant/ | 1154675 |
| 19 | child*.mp. | 2497627 |
| 20 | P?ediatric*.mp. | 425102 |
| 21 | adol*.mp. | 2147352 |
| 22 | Infan*.mp. | 1309864 |
| 23 | Teen*.mp. | 31770 |
| 24 | Youth*.mp. | 85808 |
| 25 | Preschool*.mp. | 938639 |
| 26 | Toddler*.mp. | 11834 |
| 27 | 15 or 16 or 17 or 18 or 19 or 20 or 21 or 22 or 23 or 24 or 25 or 26 | 4216287 |
| 28 | exp Dexmedetomidine/ | 3837 |
| 29 | exp Adrenergic alpha-Agonists/ | 162566 |
| 30 | dexmedetomidine.mp. | 6610 |
| 31 | sedat*.mp. | 80343 |
| 32 | Adrenergic alpha-agonists.mp. | 11027 |
| 33 | 28 or 29 or 30 or 31 or 32 | 239067 |
| 34 | 14 and 27 and 33 | 4724 |

**B. CENTRAL (January 25, 2021)**

| **Line** | **Search** | **Result** |
| --- | --- | --- |
| 1 | MeSH descriptor: [Critical Care] explode all trees | 2071 |
| 2 | MeSH descriptor: [Critical Illness] explode all trees | 2295 |
| 3 | MeSH descriptor: [Intensive Care Units] explode all trees | 3633 |
| 4 | MeSH descriptor: [Respiration, Artificial] explode all trees | 6248 |
| 5 | MeSH descriptor: [Adrenergic alpha-Agonists] explode all trees | 1132 |
| 6 | MeSH descriptor: [Dexmedetomidine] explode all trees | 1756 |
| 7 | MeSH descriptor: [Pediatrics] explode all trees | 677 |
| 8 | MeSH descriptor: [Child] explode all trees | 56050 |
| 9 | MeSH descriptor: [Adolescent] explode all trees | 104026 |
| 10 | MeSH descriptor: [Infant] explode all trees | 32106 |
| 11 | (acute care):ti,ab,kw | 23795 |
| 12 | (Critical care):ti,ab,kw | 10343 |
| 13 | (Critical* ill*):ti,ab,kw | 9625 |
| 14 | (ICU):ti,ab,kw | 13003 |
| 15 | (PICU):ti,ab,kw | 770 |
| 16 | (Intensive care):ti,ab,kw | 29159 |
| 17 | (Intubat*):ti,ab,kw | 18968 |
| 18 | (Ventilat*):ti,ab,kw | 33025 |
| 19 | (Respiratory care unit*):ti,ab,kw | 5969 |
| 20 | (Child*):ti,ab,kw | 159009 |
| 21 | (P?ediatric*):ti,ab,kw | 34719 |
| 22 | (Adol*):ti,ab,kw | 137898 |
| 23 | (Infan*):ti,ab,kw | 63378 |
| 24 | (Teen*):ti,ab,kw | 2788 |
| 25 | (Youth*):ti,ab,kw | 7520 |
| 26 | (Preschool*):ti,ab,kw | 38841 |
| 27 | (Toddler*):ti,ab,kw | 1758 |
| 28 | (Dexmedetomidine):ti,ab,kw | 5217 |
| 29 | (Adrenergic alpha-agonist*):ti,ab,kw | 888 |
| 30 | (Sedat*):ti,ab,kw | 24312 |
| 31 | 1 or 2 or 3 or 4 or 11 or 12 or 13 or 14 or 15 or 16 or 17 or 18 or 19 | 96349 |
| 32 | 7 or 8 or 9 or 10 or 20 or 21 or 22 or 23 or 24 or 25 or 26 or 27 | 285199 |
| 33 | 5 or 6 or 28 or 29 or 30 | 27670 |
| 34 | 31 and 32 and 33 | 1187 |

**C. EMBASE (January 25, 2021)**

| **Line** | **Search** | **Result** |
| --- | --- | --- |
| 1 | exp intensive care/ | 726411 |
| 2 | exp intensive care unit/ | 205845 |
| 3 | exp critical illness/ | 30720 |
| 4 | exp artificial ventilation/ | 213080 |
| 5 | Acute care.mp. | 32831 |
| 6 | Critical care.mp. | 58227 |
| 7 | Critical* ill*.mp. | 101148 |
| 8 | ICU.mp. | 125283 |
| 9 | PICU.mp. | 11153 |
| 10 | Intensive care.mp. | 373364 |
| 11 | Intubat*.mp. | 132049 |
| 12 | Ventilat*.mp. | 333231 |
| 13 | Respiratory care unit*.mp. | 328 |
| 14 | exp child/ | 2692353 |
| 15 | exp pediatrics/ | 110290 |
| 16 | exp adolescent/ | 1563419 |
| 17 | Child*.mp. | 2758095 |
| 18 | P?ediatric*.mp. | 658843 |
| 19 | Adol*.mp. | 1668537 |
| 20 | Infan*.mp. | 927961 |
| 21 | Teen*.mp. | 43527 |
| 22 | Youth*.mp. | 99832 |
| 23 | Preschool*.mp. | 569928 |
| 24 | Toddler*.mp. | 15655 |
| 25 | 14 or 15 or 16 or 17 or 18 or 19 or 20 or 21 or 22 or 23 or 24 | 4260720 |
| 26 | exp dexmedetomidine/ | 12227 |
| 27 | Dexmedetomidine.mp. | 12565 |
| 28 | Sedat*.mp. | 132578 |
| 29 | 1 or 2 or 3 or 4 or 5 or 6 or 7 or 8 or 9 or 10 or 11 or 12 or 13 | 1175530 |
| 30 | 26 or 27 or 28 | 138830 |
| 31 | 25 and 29 and 30 | 8519 |

**D. Web of Science (January 25, 2021)**

| **Line** | **Search** | **Result** |
| --- | --- | --- |
| 1 | TI=(Acute care OR Critical care OR Critical* ill* OR ICU OR PICU OR Intensive care OR Intubat* OR Ventilat* OR Respiratory care unit*) | 196183 |
| 2 | AB=(Acute care OR Critical care OR Critical* ill* OR ICU OR PICU OR Intensive care OR Intubat* OR Ventilat* OR Respiratory care unit*) | 472200 |
| 3 | TI=(Child* OR P?ediatric* OR Adol* OR Infan* OR Teen* OR Youth* OR Preschoool* OR Toddler*) | 1374783 |
| 4 | AB=(Child* OR P?ediatric* OR Adol* OR Infan* OR Teen* OR Youth* OR Preschoool* OR Toddler*) | 1473753 |
| 5 | TI=(Dexmedetomidine OR Adrenergic alpha-agonist* OR Sedat*) | 21513 |
| 6 | AB=(Dexmedetomidine OR Adrenergic alpha-agonist* OR Sedat*) | 45965 |
| 7 | 1 or 2 | 573941 |
| 8 | 3 or 4 | 2137889 |
| 9 | 5 or 6 | 55524 |
| 10 | 7 and 8 and 9 | 1715 |

**E. CINAHL (January 25, 2021)**

| **Line** | **Search** | **Result** |
| --- | --- | --- |
| 1 | (MH “Critical Care+”) | 29676 |
| 2 | (MH “Intensive Care Units+”) | 62741 |
| 3 | (MH “Respiration, Artificial+”) | 34006 |
| 4 | (MH “Critical Illness”) | 13036 |
| 5 | (MH “Critically Ill Patients”) | 12890 |
| 6 | “acute care” | 23140 |
| 7 | “critical care” | 56861 |
| 8 | “Critical* ill*” | 37657 |
| 9 | “ICU” | 38000 |
| 10 | “PICU” | 4581 |
| 11 | “intensive care” | 107500 |
| 12 | “intubat*” | 26213 |
| 13 | “ventilat*” | 55633 |
| 14 | “Respiratory care unit*” | 220 |
| 15 | 1 or 2 or 3 or 4 or 5 or 6 or 7 or 8 or 9 or 10 or 11 or 12 or 13 or 14 | 232859 |
| 16 | (MH “Pediatrics+”) | 21406 |
| 17 | (MH “Child+”) | 678656 |
| 18 | (MH “Adolescence+”) | 538313 |
| 19 | “child*” | 814024 |
| 20 | “P?ediatric*” | 26234 |
| 21 | “Adol*” | 571817 |
| 22 | “Infan*” | 441619 |
| 23 | “Teen*” | 19389 |
| 24 | “Youth*” | 51943 |
| 25 | “Preschool*” | 216936 |
| 26 | “Toddler*” | 7094 |
| 27 | 16 or 17 or 18 or 19 or 20 or 21 or 22 or 23 or 24 or 25 or 26 | 1238519 |
| 28 | (MH “Adrenergic Alpha-Antagonists+”) | 2269 |
| 29 | “dexmedetomidine” | 1955 |
| 30 | “Adrenergic alpha-agonist*” | 1542 |
| 31 | “Sedat*” | 23472 |
| 32 | 28 or 29 or 30 or 31 | 27529 |
| 33 | 15 and 27 and 32 | 1608 |

**F. Clinicaltrials.gov (January 25, 2021)**

| **Line** | **Search** | **Result** |
| --- | --- | --- |
| 1 | Advanced search  Other Terms: intensive care OR critical care OR critical illness OR  ICU OR PICU  Age group: Child (birth-17)  Intervention: Dexmedetomidine | 57 |

**G. PICUtrials.net (January 25, 2021)**

| **Line** | **Search** | **Result** |
| --- | --- | --- |
| 1 | Indication: CNS-Sedation/Analgesia | 50 |
| 2 | Intervention: Dexmedetomidine | 6 |

# Online Resource 2. Risk of bias summary

| **Study** | **Randomization process** | **Deviations from intended interventions** | **Missing data** | **Outcome measurement** | **Data reporting** | **Overall** |
| --- | --- | --- | --- | --- | --- | --- |
| **Sedation target** | | | | | | |
| Erickson 2020 | Low | High | Low | High | Low | **High** |
| Gulla 2021 | Low | Low | Low | Some concerns | Low | **Some concerns** |
| Long 2023 | Low | Low | Low | Low | High | **High** |
| **Sedation burden** | | | | | | |
| Tobias 2004 | Some concerns | Some concerns | Low | Some concerns | Some concerns | **High** |
| Garisto 2018 | Low | Some concerns | Low | Low | Low | **Some concerns** |
| Erickson 2020 | Low | High | Low | Low | Some concerns | **High** |
| Mondardini 2022 | Low | Low | Low | Low | Low | **Low** |
| Long 2023 | Low | Low | Low | Low | Low | **Low** |
| **Delirium** | | | | | | |
| Aydogan 2013 | Low | Some concerns | Low | Some concerns | Low^a^ | **Some concerns** |
| Erickson 2020 | Low | High | Low | Some concerns | Low | **High** |
| Long 2023 | Low | Low | Low | Low | Some concerns | **Some concerns** |
| **Withdrawal** | | | | | | |
| Erickson 2020 | Low | High | Low | Some concerns | Low | **High** |
| Mondardini 2022 | Low | Low | Low | Low | Low | **Low** |
| Long 2023 | Low | Low | Low | Low | Low | **Low** |
| **Duration of mechanical ventilation** | | | | | | |
| Aydogan 2013 | Low | Some concerns | Low | Low | Some concerns | **Some concerns** |
| Garisto 2018 | Low | Some concerns | Low | Low | Low | **Some concerns** |
| Erickson 2020 | Low | High | Low | Low | Low | **High** |
| Gulla 2021 | Low | Low | Low | Low | Low | **Low** |
| Becker 2023 | Low | Low | Low | Low | Low | **Low** |
| Long 2023 | Low | Low | Low | Low | Low | **Low** |
| **Time to extubation** | | | | | | |
| Prasad 2012 | Some concerns | Low | Low | Low | Some concerns | **Some concerns** |
| Husssein 2013 | Some concerns | Low | Low | Low | High | **High** |
| Saleh 2016 | Some concerns | Low | Low | Low | Some concerns | **Some concerns** |
| Attia 2022 | Some concerns | Some concerns | Low | Low | Some concerns | **High** |
| **PICU length of stay** | | | | | | |
| Aygodan 2013 | Low | Some concerns | Low | Low | Some concerns | **Some concerns** |
| Garisto 2018 | Low | Some concerns | Low | Low | Low | **Some concerns** |
| Erickson 2020 | Low | Some concerns | Low | Low | Low | **Some concerns** |
| Gulla 2021 | Low | Low | Low | Low | Low | **Low** |
| Mondardini 2022 | Low | Low | Low | Low | Low | **Low** |
| Becker 2023 | Low | Low | Low | Low | Low | **Low** |
| Long 2023 | Low | Low | Low | Low | Low | **Low** |
| **Hospital length of stay** | | | | | | |
| Erickson 2020 | Low | Some concerns | Low | Low | Low | **Some concerns** |
| Gulla 2021 | Low | Low | Low | Low | Low | **Low** |
| Long 2023 | Low | Low | Low | Low | Low | **Low** |
| **Adverse events** |  |  |  |  |  |  |
| Tobias 2004 | Some concerns | Some concerns | Low | Low | Some concerns | **High** |
| Prasad 2012 | Some concerns | Low | Low | Low | High | **High** |
| Aygodan 2013 | Low | Some concerns | Low | Low | Some concerns | **Some concerns** |
| Hussein 2013 | Some concerns | Low | Low | Low | Some concerns | **Some concerns** |
| Saleh 2016 | Some concerns | Low | Low | Low | High | **High** |
| Garisto 2018 | Low | Some concerns | Low | Low | Low | **Some concerns** |
| Erickson 2020 | Low | High | Low | Low | Low | **High** |
| Gulla 2021 | Low | Low | Low | Low | Low | **Low** |
| Mondardini 2022 | Low | Low | Low | Low | Low | **Low** |
| Becker 2023 | Low | Low | Low | Low | Low | **Low** |
| Long 2023 | Low | Low | Low | Low | Low | **Low** |

Legend: ^a^ This author was contacted regarding the reported results for this outcome and provided clarified data. This domain assessment was changed from an initial evaluation of “some concerns” to “low,” which also resulted in a change of overall risk of bias assessment for this outcome from “high” to “some concerns.”

# Online Resource 3. Evidence profile

| **Certainty assessment** | | | | | | | **№ of patients** | | **Effect** | | **Certainty** |
| --- | --- | --- | --- | --- | --- | --- | --- | --- | --- | --- | --- |
| **No. of studies** | **Study design** | **Risk of bias** | **Inconsistency** | **Indirectness** | **Imprecision** | **Other**  **considerations** | **Dexmedetomidine** | **Control** | **Relative (95% CI)** | **Absolute (95% CI)** |  |
| **Sedation target** | | | | | | | | | | | |
| 3 | randomised trials | serious^a^ | serious^b^ | not serious^c^ | serious^d^ | none | Sedation efficacy was assessed in three trials (n=170 participants). Two trials (Erickson 2020, Long 2023) found that dexmedetomidine, compared to other sedatives, increased the portion of sedation assessments within sedation target. However, CIs of the effect estimate in one trial (Long 2023) included both substantially higher, and a possibility of less, assessments within sedation target. The remaining trial (Gulla 2021) found that dexmedetomidine, compared to midazolam, decreased time and proportion of assessments within sedation target. | | | | ⨁◯◯◯ Very low^a,b,c,d^ |
| **Duration of mechanical ventilation** | | | | | | | | | | | |
| 6 | randomised trials | not serious | not serious | not serious^e^ | serious^f^ | none | 148 | 132 | - | MD **2.18 hours lower** (3.28 lower to  1.09 lower) | ⨁⨁⨁◯ Moderate^e,f^ |
| **Time to extubation** | | | | | | | | | | | |
| 4 | randomised trials | serious^g^ | serious^h^ | not serious | serious^i^ | none | 110 | 110 | - | MD **2.99 hours lower** (4.88 lower to  1.11 lower) | ⨁◯◯◯ Very low^g,h,i^ |
| **Delirium** | | | | | | | | | | | |
| 3 | randomised trials | serious^g^ | not serious | not serious | serious^j^ | none | 29/68 (42.6%) | 33/58 (56.9%) | **RR 0.83** (0.64 to 1.07) | **97 fewer per 1,000** (from 205 fewer to  40 more) | ⨁⨁◯◯ Low^g,j^ |
| **Withdrawal** | | | | | | | | | | | |
| 3 | randomised trials | serious^g^ | not serious | not serious | very serious^k^ | none | 19/81 (23.5%) | 23/82 (28.0%) | **RR 0.93** (0.55 to 1.59) | **20 fewer per 1,000** (from 126 fewer to 165 more) | ⨁◯◯◯ Very low^g,k^ |
| **Bradycardia** | | | | | | | | | | | |
| 8 | randomised trials | not serious^l^ | not serious | serious^m^ | serious^n^ | none | 31/181 (17.1%) | 7/164 (4.3%) | **RR 2.45** (0.90 to 6.65) | **62 more per 1,000** (from 4 fewer to  241 more) | ⨁⨁◯◯ Low^l,m,n^ |
| **Hypotension** | | | | | | | | | | | |
| 9 | randomised trials | not serious^l^ | not serious | serious^o^ | serious^p^ | none | 20/236 (8.5%) | 4/209 (1.9%) | **RR 1.99** (0.80 to 4.96) | **19 more per 1,000** (from 4 fewer to  76 more) | ⨁⨁◯◯ Low^l,o,p^ |
| **Clinically important bradycardia** | | | | | | | | | | | |
| 10 | randomised trials | not serious^q^ | not serious | not serious | serious^f^ | none | 13/255 (5.1%) | 3/228 (1.3%) | **RR 1.42** (0.45 to 4.49) | **6 more per 1,000** (from 7 fewer to  46 more) | ⨁⨁⨁◯ Moderate^f,q^ |
| **Clinically important hypotension** | | | | | | | | | | | |
| 8 | randomised trials | not serious^r^ | not serious | not serious | serious^f^ | none | 13/206 (6.3%) | 3/179 (1.7%) | **RR 1.35** (0.48 to 3.82) | **6 more per 1,000** (from 9 fewer to  47 more) | ⨁⨁⨁◯ Moderate^f,r^ |
| **PICU length of stay** | | | | | | | | | | | |
| 7 | randomised trials | not serious | not serious | not serious^s^ | serious^t^ | none | 166 | 154 | - | MD **0.2 days fewer** (0.35 fewer to  0.05 fewer) | ⨁⨁⨁◯ Moderate^s,t^ |
| **Hospital length of stay** | | | | | | | | | | | |
| 3 | randomised trials | not serious | not serious | not serious | very serious^u^ | none | 86 | 84 | - | MD **0.53 days more** (2.97 fewer to  4.03 more) | ⨁⨁◯◯ Low^u^ |

CI: confidence interval; MD: mean difference; RR: risk ratio

#### Explanations

a. All three trials have important risk of bias concerns (2 high ROB, 1 some concerns for ROB).

b. The direction and magnitude of effect varied across the different trials. Two trials found that dexmedetomidine, compared to other sedatives, increased the portion of sedation assessments within sedation target. The remaining trial found that dexmedetomidine, compared to midazolam, decreased time and proportion of assessments within sedation target.

c. The patients, intervention and comparators in the included trials all provide direct evidence to the clinical question at hand. We did not downgrade for indirectness considering one trial did not use a validated sedation scale because the certainty of evidence is already very low.

d. Small sample size (n=170). Trials reported variable magnitude of positive effect (higher proportion of sedation assessments within target), with one trial having a wide CI including substantially higher, and a possibility of less, assessments within sedation target. One trial reported decreased time and proportion of assessments within sedation target.

e. One trial (contributing the large majority of weight to the meta-analysis) enrolled participants post-scoliosis surgery with a very short duration of mechanical ventilation and PICU length of stay, in both intervention and control groups. Post-hoc sensitivity analyses excluding this trial did not impact the effect estimate and confidence interval to include a minimal clinically important difference (MD 0.75 hours; 95% CI -13.9, 15.4).

f. The point estimate is consistent with a trivial to no effect, and the upper and lower limits of the CI are also trivial/no effect. However, the overall sample size is small and below optimal information size.

g. All trials contributing data to the analysis have high risk of bias.

h. I2 = 97% (p < 0.001). Three trials demonstrate an important reduction in time to extubation (~3-4 hours) while one trial's result is a trivial/small benefit only (54 minute reduction). Unable to explain inconsistency by trial population (all surgical patients) or risk of bias (no low ROB studies).

i. The overall sample size is small and below optimal information size.

j. Wide CI that includes includes meaningful benefit, to small/no effect. Sample size is below optimal information size.

k. Rated down 2 for imprecision considering the effect estimate CI is wide and includes important benefit and important harm.

l. Most studies at high risk of bias or some concerns for risk of bias; however, no significant subgroup effect was found.

m. Importantly variable definitions of bradycardia, including some trials that did not report their case definition.

n. Rated down for imprecision considering upper end of CI includes harm. The overall event rate is also low.

o. Importantly variable definitions of hypotension, including some trials that did not report their case definition.

p. Rated down for imprecision as sample size is below optimal information size.

q. Analysis included studies with some concerns/high ROB, but no significant subgroup effect was found.

r. While the majority of studies have some concerns/high ROB, they report no overall events and do not contribute to the effect estimate. The studies contributing to the effect estimate have low ROB for this outcome.

s. One trial (contributing the large majority of weight to the meta-analysis) enrolled participants post-scoliosis surgery with a very short duration of mechanical ventilation and PICU length of stay, in both intervention and control groups. Post-hoc sensitivity analyses excluding this trial did not impact the effect estimate and confidence interval to include a minimal clinically important difference (MD -0.28 days; 95% CI -1.33, 0.77).

t. The effect estimate of PICU length of stay is approximately a reduction of 5 hours (a trivial effect), and the CI does not include substantial benefit (as much as an 8 hour reduction) or harm (as much as a 1 hour reduction). However, the overall sample size is small and below optimal information size.

u. The point estimate is consistent with a trivial effect (~12 hours increased hospital length of stay) and the CI includes the possibility of both important benefit (~3 days less) and important harm (~4 days more); therefore, we rated down 2 for imprecision. The effect estimate considers data from a very small overall sample size.

# Online Resource 4. Sedation target outcome analysis*^a^*

| **Source** | **Sedation Target** | **Summary Measure** | **Intervention** | **Control** | **Estimate of Effect (95% CI)***^b^* | **Risk of bias** |
| --- | --- | --- | --- | --- | --- | --- |
| Erickson 2020  n=57 | SBS -1 to +1 (light sedation) | Assessments within sedation target, n (%) | | | | High |
|  |  | a. First 24h | 66/103 (64%) | 48/116 (41%) | 2.5 (9.8, 50.3) |  |
|  |  | b. First 48h | 229/325 (71%) | 181/311 (58%) | 2.20 (1.03, 4.72) |  |
| Gulla 2021  n=47 | PSCH sedation scale level 4 or 5  (moderate to deep sedation) | Time (hrs) in sedation target, median (IQR) | 20 (6, 28) | 38 (20.5, 66) | p=0.006 | Some concerns |
|  |  | Assessments (%) within sedation target, mean (SD) | 56.5 (28.6) | 67.3 (18.8) | Mean difference  -10.9 (-25.15, 3.25) |  |
| Long 2023  n=66 | SBS -1 to +1  (light sedation) | Assessments within sedation target, n (%) | | | | High |
|  |  | a. First 24h | 76 (44.7%) | 60 (33.7%) | 4.14 (0.48, 35.92) |  |
|  |  | b. First 48h | 154 (51.7%) | 122 (38.9%) | 6.95 (0.77, 63.13) |  |
|  |  | c. Total study duration | 597 (71.8%) | 527 (56.1%) | 3.93 (0.62, 25.03) |  |

Legend: *^a^*Results describe proportion of sedation scores within study protocol sedation target. *^b^*Odds ratio (95% CI), unless reported otherwise. CI, indicates confidence interval; IQR, interquartile range; NSS, not statistically significant; PSCH, Penn State Children Hospital; SBS, State Behavioural Scale; SD, standard deviation.

# Online Resource 5. Sedation burden outcome analysis*^a^*

| **Source** | **Sedative Exposure Measured** | **Intervention** | **Control** | **Estimate of Effect***^b^* | **Risk of bias** |
| --- | --- | --- | --- | --- | --- |
| Long 2023  n=66 | Dexmedetomidine | 16.2 (9.5, 41.9) | 3.4 (1.9, 18.1) | NR | Low |
|  | Midazolam | 720.0 (203.6, 1260.7) | 2503.2 (1160.6, 6760.5) | NR |  |
|  | Midazolam rescue | 123.3 (74.4, 200.0) | 100.0 (74.5, 177.9) | NR |  |
|  | Morphine | 1459.2 (711.2, 3866.7) | 2258.2 (1197.8, 4896.2) | NR |  |
|  | Fentanyl | 44.6 (24.5, 72.8) | 45.8 (38.9, 73.6) | NR |  |
|  | Chloral hydrate (mg/kg) | 30.7 (12.0, 96.2) | 57.1 (15.3, 110.2) | NR |  |
|  | Ketamine | 937.5 (500.0, 1943.4) | 759.6 (500.0, 2857.1) | NR |  |
| Mondardini 2022  n=40 | Opioid (fentanyl equivalents) | 490 (378.4, 688.0) | 630 (492.0, 893.0) | p=0.103 | Low |
|  | Midazolam (mg/kg) | 57.2 (27.7, 93.9) | 60.3 (36.2, 99.4) | p=0.870 |  |
| Erickson 2020  n=47 | Dexmedetomidine | 47.8 (35.87, 70.97) | 3.51 (2.96, 4.05) | p=0.005 | High |
|  | Midazolam (mg/kg) | 0.17 (0.09, 0.56) | 2.72 (1.16, 5.10) | p=0.002 |  |
|  | Propofol (mg/kg) | 58 (15.65, 67.53) | 15.42 (8.50, 49.25) | p=0.3 |  |
|  | Ketamine (mg/kg) | 0.84 (0.36, 6.21) | 0.12 (0.12, 0.12) | p=0.4 |  |
|  | Morphine (mg/kg) | 1.90 (1.12, 3.96) | 1.55 (1.11, 2.34) | p=0.3 |  |
|  | Fentanyl | 42.2 (25.73, 83.54) | 21.49 (6.55, 53.42) | p=0.4 |  |
| Garisto 2018  n=48 | Morphine (mg/kg) | 0.79 (0.62, 1.12) | 0.76 (0.55, 1.4) | p=0.92 | Some concerns |
|  | Midazolam (mg/kg) | 2.97 (1.1, 4.6) | 2.77 (1.4, 6.6) | p=0.67 |  |
| Tobias 2004  n=30 | Morphine (mg/kg), mean (SD) | *0.25 Dexmedetomidine Group*  0.55 (0.38) | 0.74 (0.5) | p=NSS | High |
|  |  | *0.5 Dexmedetomidine Group*  0.28 (0.12) |  | p=0.01 |  |

Legend: *^a^*Results describe median (Q1, Q3) cumulative weight-adjusted sedative dose for total study period in mcg/kg, unless otherwise specified. *^b^*As reported by authors. NR indicates not reported; NSS, not statistically significant; SD, standard deviation.

# Online Resource 6. Forest plots

**A.** Duration of mechanical ventilation (hours) forest plot


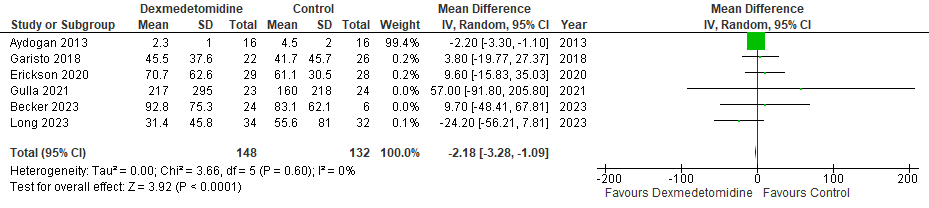


**B.** Time to extubation (hours) forest plot


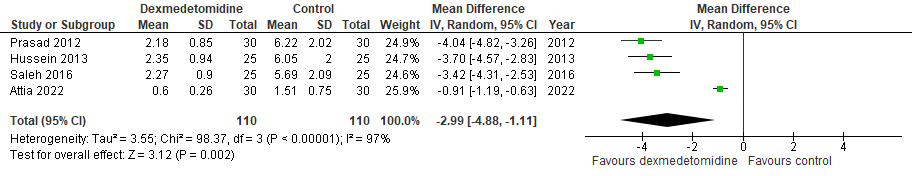


**C.** Delirium forest plot


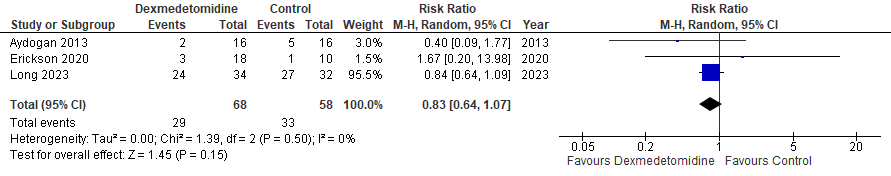


**D.** Withdrawal forest plot


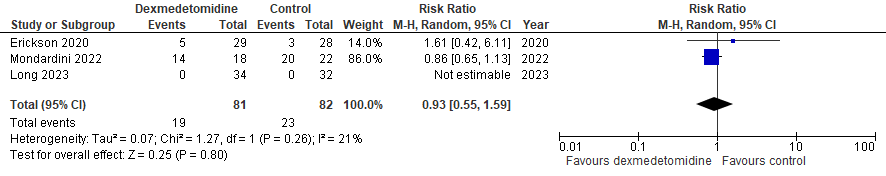


**E.** Bradycardia forest plot


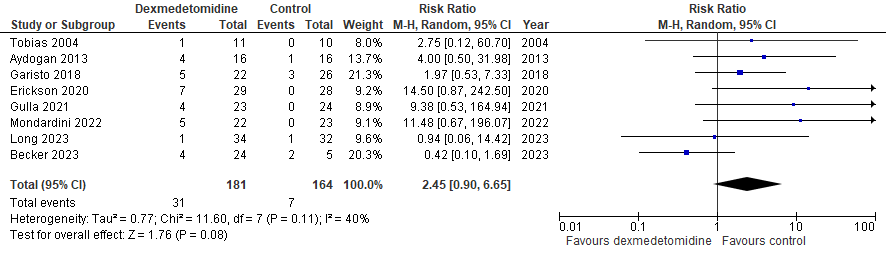


**F.** Hypotension forest plot


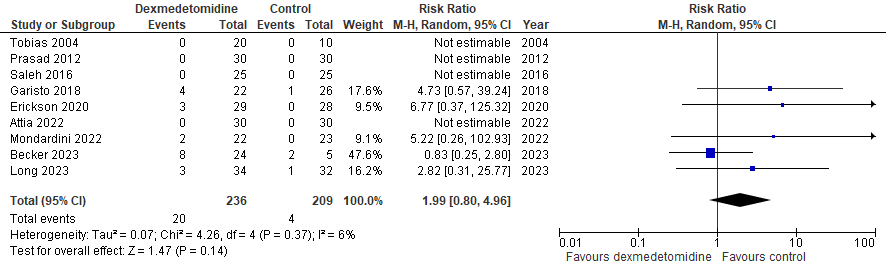


**G.** Clinically important bradycardia forest plot


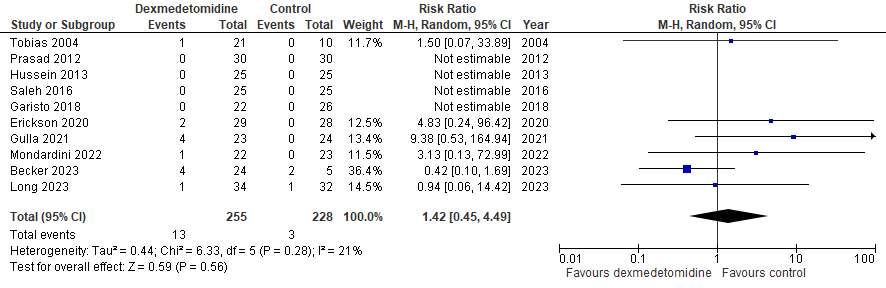


**H.** Clinically important hypotension forest plot


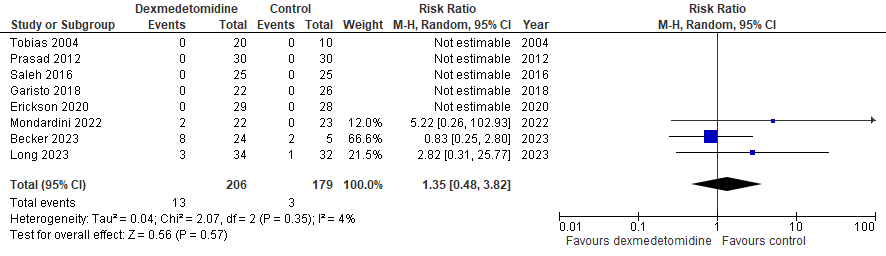


**I.** PICU length of stay (days) forest plot


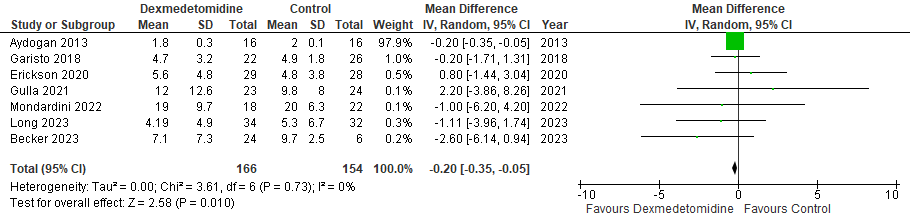


**J.** Hospital length of stay (days) forest plot


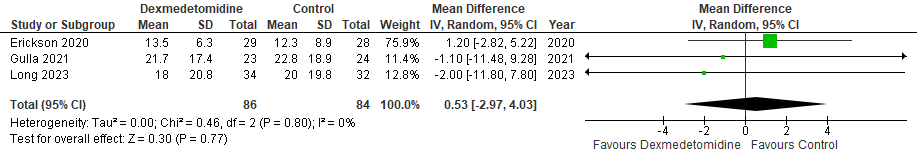


# Online Resource 7. Summarized author definitions of adverse events

| **Source** | **Hypotension** | **Clinically important hypotension** | **Bradycardia** | **Clinically important bradycardia** |
| --- | --- | --- | --- | --- |
| **Tobias et al.**  **2004** | NR | Study drug discontinuation | NR | Study drug discontinuation |
| **Prasad et al.**  **2012** | NR | NR | n/a | Study drug dose reduction, discontinuation, pharmacological intervention, or temporary pacing |
| **Aydogan et al.**  **2013** | n/a | n/a | ≥20% decrease from baseline pre-operative value | n/a |
| **Hussein et al.**  **2013** | n/a | n/a | n/a | Study drug dose reduction or discontinuation |
| **Saleh et al.**  **2016** | NR | Study drug dose reduction or discontinuation | n/a | Study drug dose reduction or discontinuation |
| **Garisto et al.**  **2018** | ≥20% decrease in mean arterial BP | Pharmacological intervention | ≥20% decrease from baseline value | Pharmacological intervention |
| **Erickson et al.**  **2020** | ≥2 SD below mean for age | Requiring intervention (e.g. study drug discontinuation) | ≥2 SD below mean for age | Requiring intervention (e.g. study drug discontinuation) |
| **Mondardini et al.**  **2021** | ≥20% decrease BP from baseline | Study drug discontinuation | ≥20% decrease from baseline value | Study drug discontinuation |
| **Gulla et al.**  **2021** | n/a | n/a | <60 beats/min | Study drug discontinuation |
| **Long et al.**  **2023** | ≥2 SD below mean for age, which lead to study drug cessation | | | |
| **Becker et al.**  **2023** | Study drug dose decrease, discontinuation, fluid administration, inotrope/vasopressor initiation or dose increase | | Study drug dose reduction, discontinuation, pharmacological intervention, or temporary pacing | |

Legend: BP indicates blood pressure; n/a, not applicable (i.e. the study did not measure this outcome); NR, definition not reported.

# Online Resource 8. Subgroup analyses

**A.** Patient population subgroup analysis: Duration of mechanical ventilation (hours)


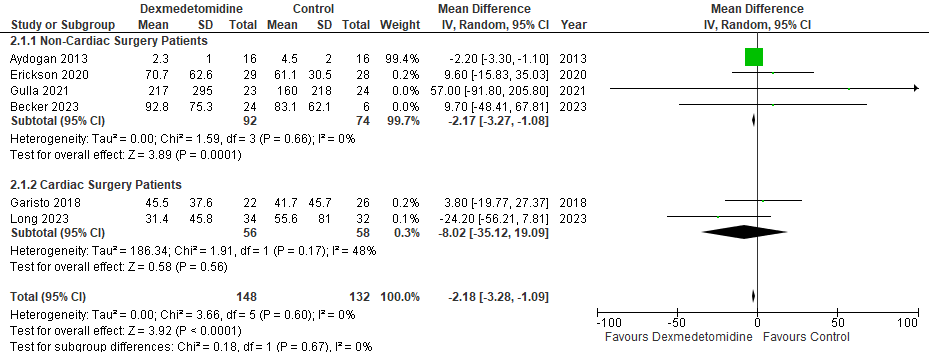


**B.** Patient population subgroup analysis: Time to extubation (hours)


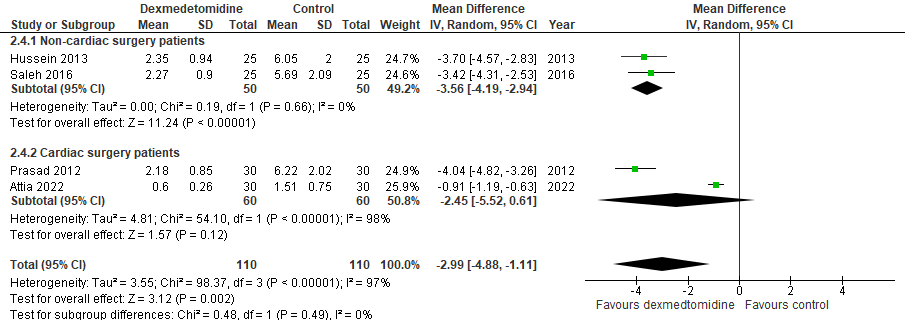


**C.** Patient population subgroup analysis: PICU length of stay (days)


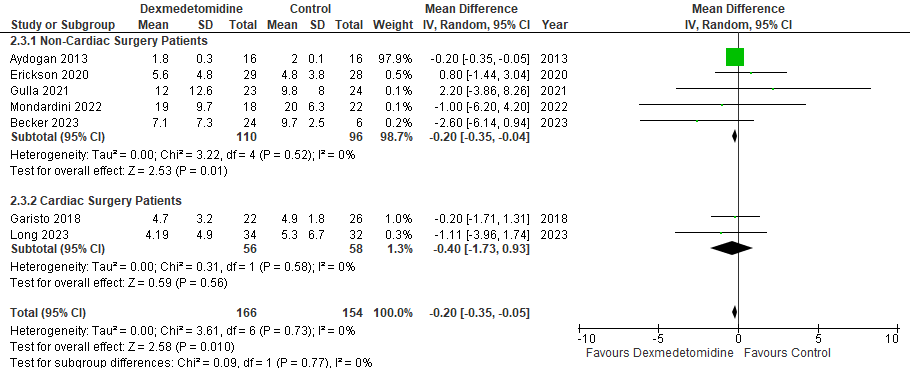


**D.** Patient population subgroup analysis: Hypotension


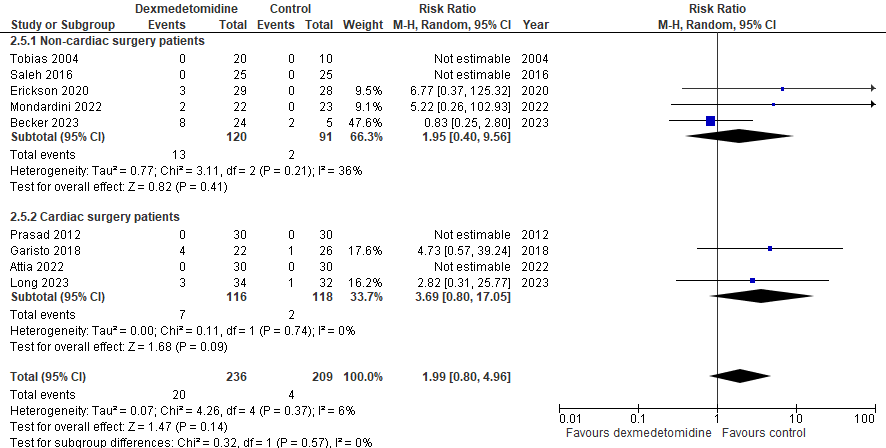


**E.** Patient population subgroup analysis: Bradycardia


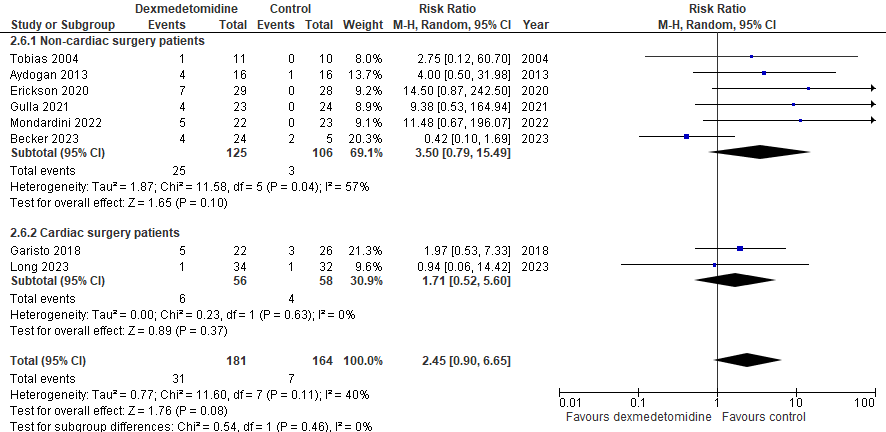


**F.** Intervention type subgroup analysis: Duration of mechanical ventilation


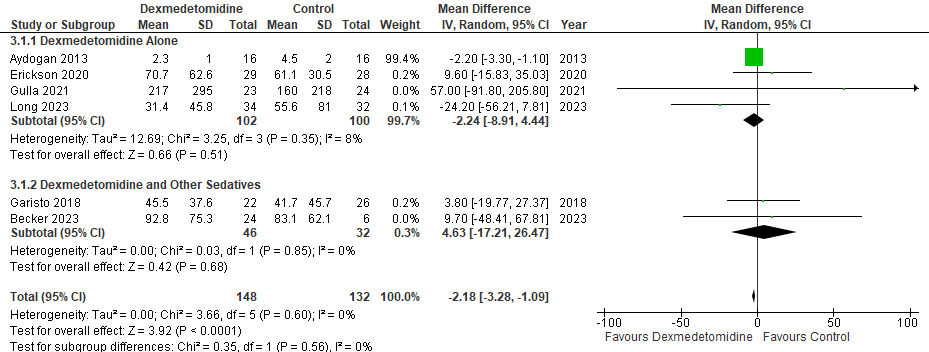


**G.** Intervention type subgroup analysis: PICU length of stay


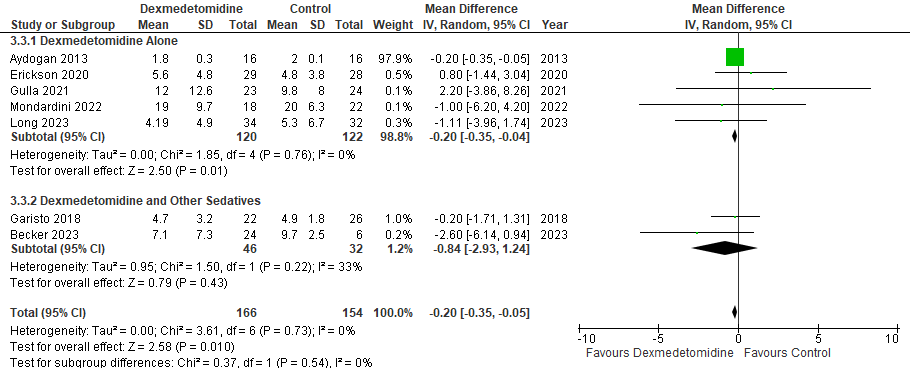


**H.** Intervention type subgroup analysis: Hypotension


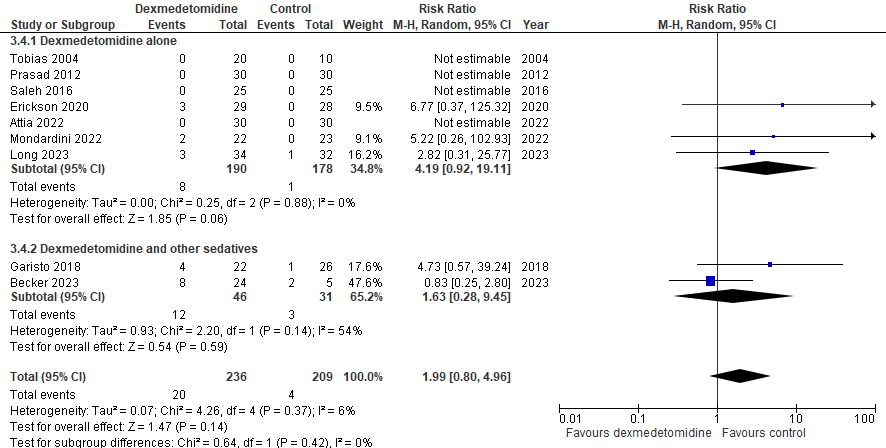


**I.** Intervention type subgroup analysis: Bradycardia


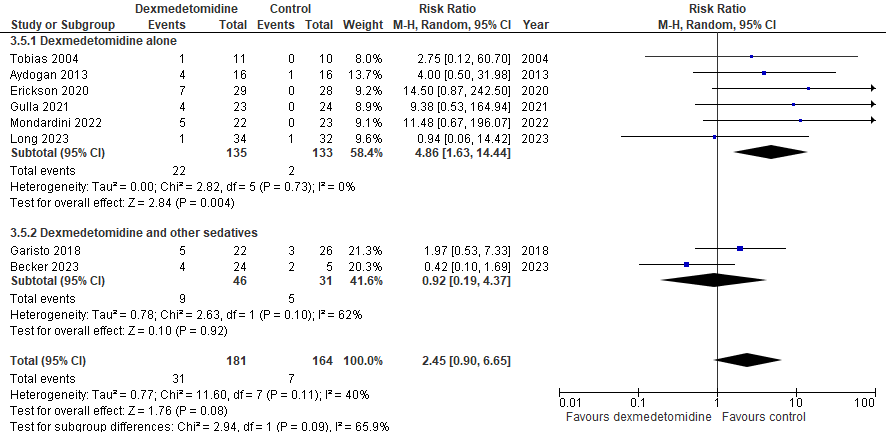


**J.** Comparator type subgroup analysis: PICU length of stay


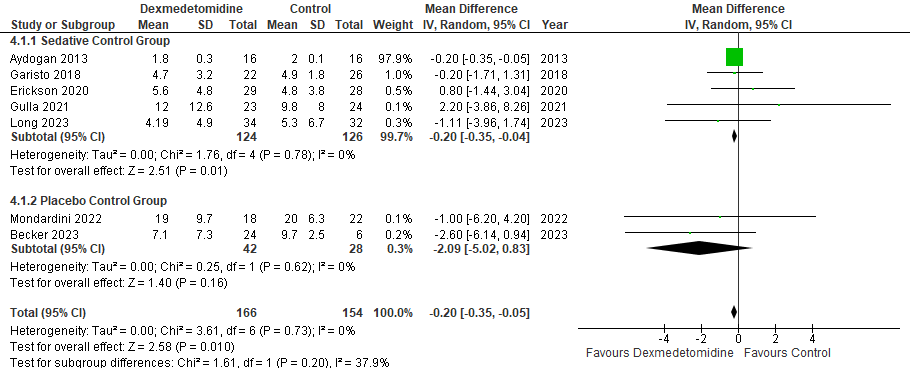


**K.** Comparator type subgroup analysis: Hypotension


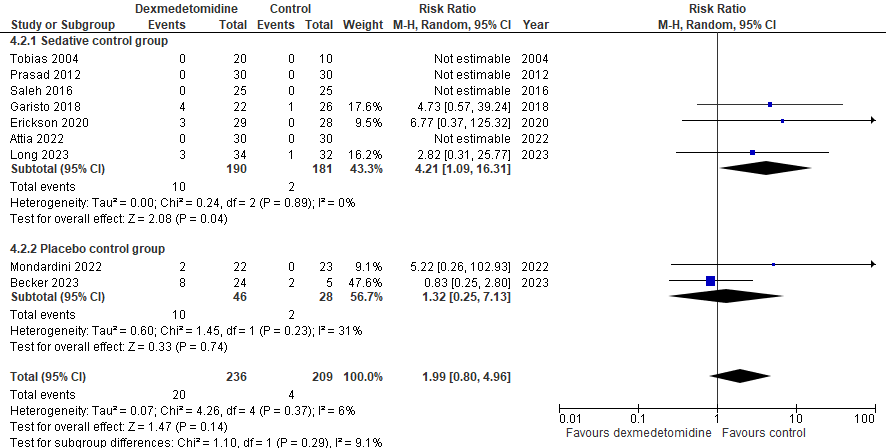


**L.** Comparator type subgroup analysis: Bradycardia


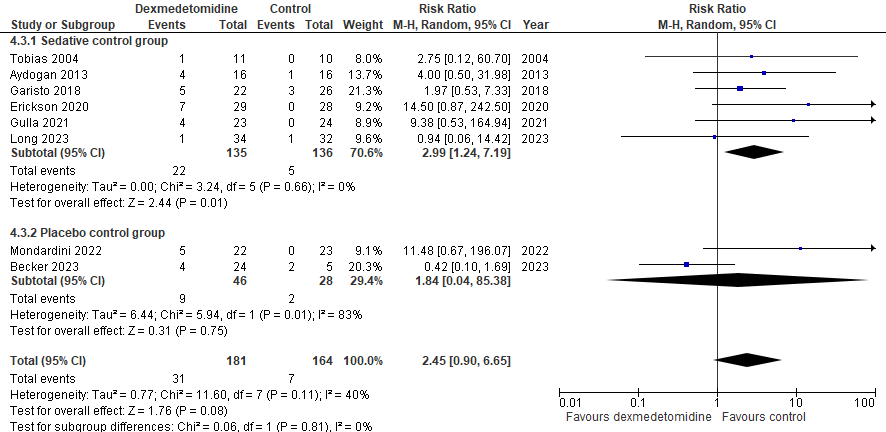


**M.** Comparator type subgroup analysis: Clinically important bradycardia


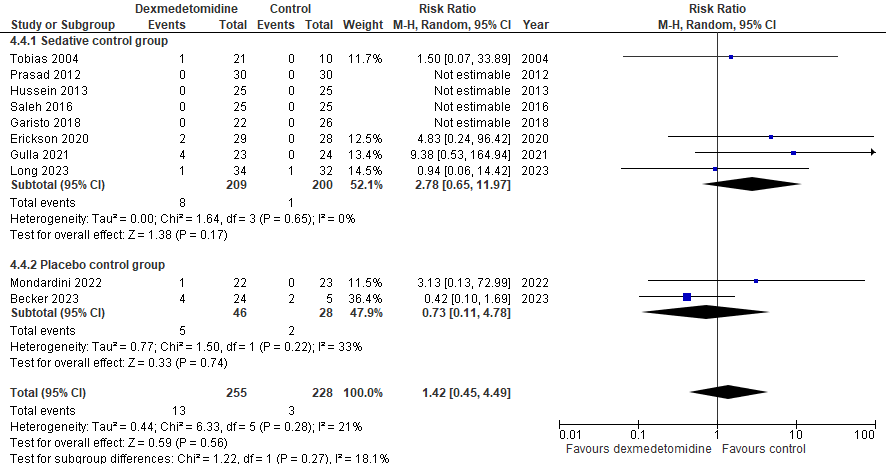


**N.** Risk of bias sensitivity analysis: Duration of mechanical ventilation


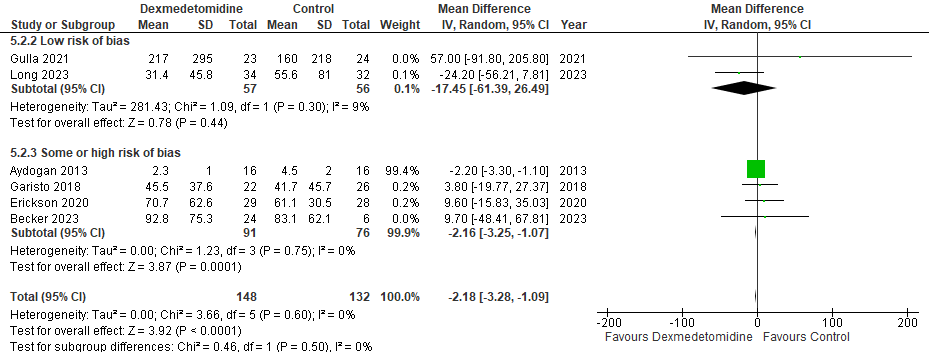


**O.** Risk of bias sensitivity analysis: PICU length of stay


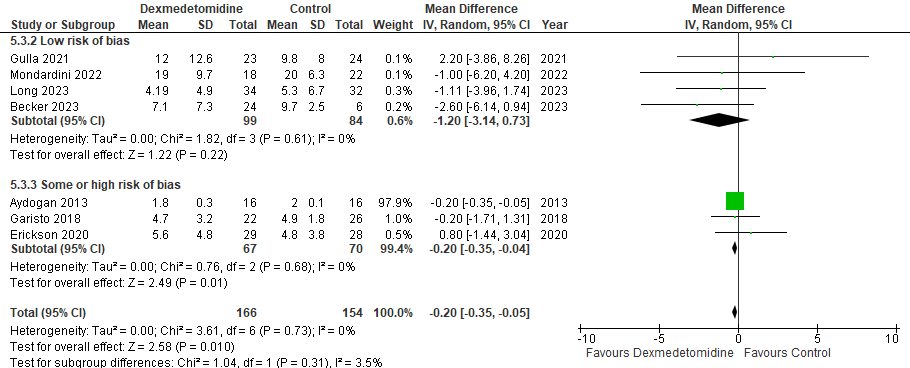


**P.** Risk of bias sensitivity analysis: Hypotension


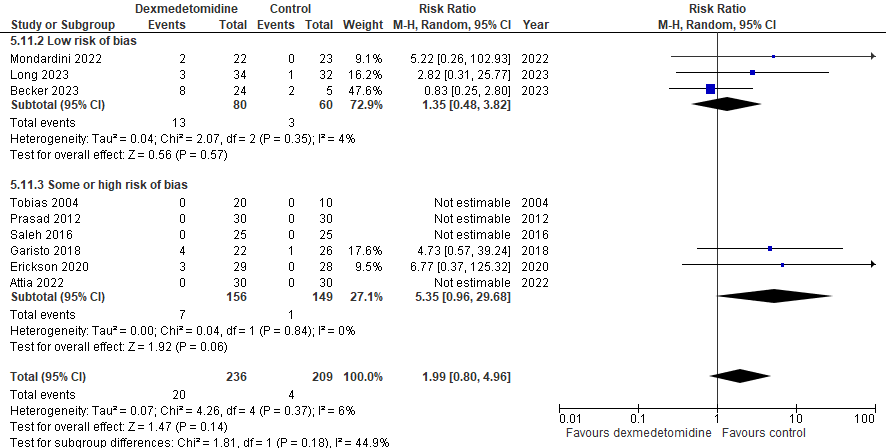


**Q.** Risk of bias sensitivity analysis: Bradycardia


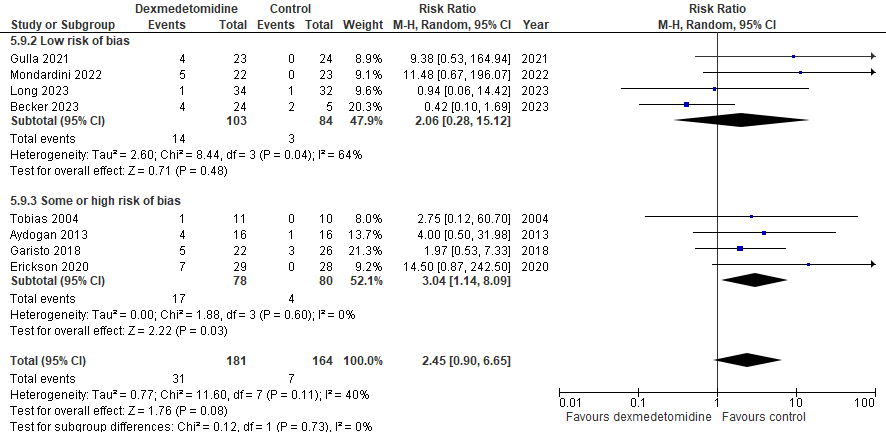


**R.** Risk of bias sensitivity analysis: Clinically important bradycardia


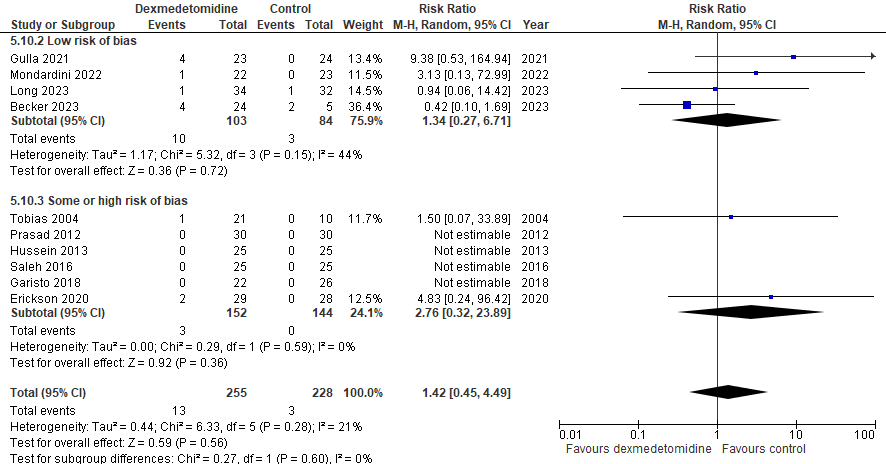

Supplement: Supplementary file 1 — Supplementary material 1. Online Resource 1. Search strategies Online Resource 2. Risk of bias summary Online Resource 3. Evidence profile Online Resource 4. Sedation target outcome narrative summary Online Resource 5. Sedation burden outcome narrative summary Online Resource 6. Forest plots Online Resource 7. Summarized author definitions of adverse events Online Resource 8. Subgroup and sensitivity analyses [file 44253_2025_91_MOESM1_ESM.docx]
